# Revealing the Electrophilic‐Attack Doping Mechanism for Efficient and Universal p‐Doping of Organic Semiconductors

**DOI:** 10.1002/advs.202203111

**Published:** 2022-09-11

**Authors:** Jing Guo, Ying Liu, Ping‐An Chen, Xinhao Wang, Yanpei Wang, Jing Guo, Xincan Qiu, Zebing Zeng, Lang Jiang, Yuanping Yi, Shun Watanabe, Lei Liao, Yugang Bai, Thuc‐Quyen Nguyen, Yuanyuan Hu

**Affiliations:** ^1^ International Science and Technology Innovation Cooperation Base for Advanced Display Technologies of Hunan Province College of Semiconductors (College of Integrated Circuits) Hunan University Changsha 410082 P. R. China; ^2^ State Key Laboratory of Chem‐/Bio‐Sensing and Chemometrics School of Chemistry and Chemical Engineering Hunan University Changsha Hunan 410082 P. R. China; ^3^ Beijing National Laboratory for Molecular Sciences Key Laboratory of Organic Solids Institute of Chemistry Chinese Academy of Sciences Beijing 100190 P. R. China; ^4^ Material Innovation Research Center (MIRC) and Department of Advanced Material Science Graduate School of Frontier Sciences The University of Tokyo 5‐1‐5 Kashiwanoha Kashiwa Chiba 77‐8561 Japan; ^5^ Center for Polymers and Organic Solids Department of Chemistry and Biochemistry University of California at Santa Barbara Santa Barbara CA 93106 USA; ^6^ Shenzhen Research Institute of Hunan University Shenzhen 518063 P. R. China

**Keywords:** cation, doping efficiency, doping mechanism, electrophilic attack, organic semiconductors

## Abstract

Doping is of great importance to tailor the electrical properties of semiconductors. However, the present doping methodologies for organic semiconductors (OSCs) are either inefficient or can only apply to some OSCs conditionally, seriously limiting their general applications. Herein, a novel p‐doping mechanism is revealed by investigating the interactions between the dopant trityl tetrakis(pentafluorophenyl) borate (TrTPFB) and poly(3‐hexylthiophene) (P3HT). It is found that electrophilic attack of the trityl cations on thiophenes results in the formation of tritylated thiophenium ions, which subsequently induce electron transfer from neighboring P3HT chains to realize p‐doping. This unique p‐doping mechanism enables TrTPFB to p‐dope various OSCs including those with high ionization energy (IE ≈ 5.8 eV). Moreover, this doping mechanism endows TrTPFB with strong doping capability, leading to doping efficiency of over 80% in P3HT. The discovery and elucidation of this novel doping mechanism not only points out that strong electrophiles are a class of efficient p‐dopants for OSCs, but also provides new opportunities toward highly efficient doping of various OSCs.

## Graphical abstract

A novel p‐doping mechanism for organic semiconductors (OSCs) based on electrophilic attack of the trityl cations is revealed. This unique p‐doping mechanism not only enables the dopant to p‐dope various OSCs including those with high ionization energy, but also endows the dopant with strong doping capability.

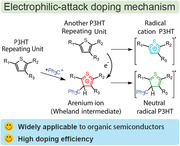

## 1 Introduction

Doping has been proven to be essential for fabricating various high‐performance organic devices including organic light‐emitting diodes,^[^ ^1^ ^]^ organic photovoltaics (OPVs),^[^ ^2^ ^]^ organic field‐effect transistors (OFETs),^[^ ^3^ ^]^ and organic thermoelectric generators (OTEGs).^[^ ^4^ ^]^ However, in contrast to doping in silicon, where a small amount of substitutional dopants can efficiently generate electrons (n‐doping) or holes (p‐doping), highly efficient and controllable doping of organic semiconductors (OSCs) is an intensively studied topic yet remains a challenge.^[^ ^1^, ^5^ ^]^

Taking p‐doping of OSC as an example, presently there are mainly two mechanisms: redox reaction and protonation. The redox reaction doping mechanism requires matching the lowest unoccupied molecular orbital (LUMO, corresponds to electron affinity (EA)) level of the dopant with the highest occupied molecular orbital (HOMO, corresponds to ionization energy (IE)) level of the host semiconductor, by which integer or partial electron transfer can occur between them.^[^ ^5^, ^6^ ^]^ This doping mechanism has been widely adopted by molecular dopants such as F_4_TCNQ, but it has a serious limitation, namely only those OSCs with HOMO levels comparable to or higher than the LUMO level of the dopant can be effectively doped (i.e., conditional doping effect). As an example, OSCs with HOMO deeper than −5.3 eV cannot be well doped by F_4_TCNQ since the LUMO of F_4_TCNQ is −5.2 eV. Although ultrahigh doping efficiency based on this doping mechanism has been reported by adopting anion exchange or double doping strategies,^[^ ^5^, ^7^ ^]^ the conditional doping effect is a significant barrier for the general application of the redox reaction doping mechanism. In addition, molecular dopants like F_4_TCNQ have low solubility in organic solvents (<2 g L^−1^ in tetrahydrofuran (THF)) and tend to aggregate in thin films even at a low doping concentration, preventing the semiconductors from being heavily doped.^[^ ^8^ ^]^

The other doping mechanism, i.e., protonation doping, relies on the protonation of arenes on OSCs by strong Brønsted acids such as trifluoroacetic acid (TFA)^[^ ^9^ ^]^ and HBr.^[^ ^10^ ^]^ Recently, this doping mechanism was found to extend to Lewis acids such as tris(pentafluorophenyl)borane (B(C_6_F_5_)_3_), which hydrolyzes to form Brønsted acids that can p‐dope OSCs. Because the polarons are generated in spontaneous electron transfer processes initiated by the arenium ions, i.e., protonated arenes, this doping mechanism seems to place no constraints on the energy levels of host OSCs. For example, B(C_6_F_5_)_3_ can p‐dope several OSCs with HOMO deeper than −5.2 eV.^[^ ^11^ ^]^ However, the doping efficiency of B(C_6_F_5_)_3_ is reported to be low when used as a dopant for P3HT (less than 20%),^[^ ^12^ ^]^ indicating the protonation doping mechanism does not promise sufficiently high doping performance.^[^ ^12^ ^]^ Although the doping physics of B(C_6_F_5_)_3_ is still in argument, one important reason accounting for its low doping efficiency is the high reversibility of arene protonation, which can significantly limit the efficiency of the electron transfer process that follows (see more details in Section S5.2, Supporting Information).

The above discussions suggest the challenges in achieving universal doping effect and high doping efficiency simultaneously in OSCs. Notably, some novel dopants with remarkable doping performance have been reported recently.^[^ ^13^ ^]^ For instance, we reported an organic salt trityl tetrakis(pentafluorophenyl) borate (TrTPFB) containing a trityl cation and the tetrakis(pentafluorophenyl) borate (TPFB) anion, which was shown to effectively p‐dope organic semiconductors.^[^ ^14^ ^]^ Wegner et al. reported a similar organic salt with borinium cation Mes_2_B‐TPFB (Mes_2_B^+^; Mes: mesitylene), and used this organic salt as a p‐dopant in P3HT.^[^ ^13c^ ^]^ The borinium salt showed an occurrence of a bipolaronic peak, suggesting the strong doping capability of the salt. In addition, these organic salt dopants are found to have good miscibility with semiconductors, which is an important property desired for dopants. In spite of the promising applications of the organic salt dopants, a systematic and in‐depth study on their doping performance is in lack, and in particular, their doping mechanism remains elusive.

In this work, we performed a series of experiments to understand the doping performance and doping mechanism of TrTPFB, by which we reveal a new p‐doping mechanism based on electrophilic attack of cations on OSCs. Specifically, we show that electrophilic attack of the trityl cation (Ph_3_C^+^) on thiophene rings leads to the tritylation and subsequent p‐doping of P3HT. Importantly, such electrophilic attack behavior is expected to universally and efficiently happen between the trityl cation and OSCs. As a result, the dopant TrTPFB is capable of p‐doping a wide range of OSCs regardless of their HOMO levels. Moreover, the dopant exhibits high doping capability by resulting in conductivity of 30 S cm^−1^ in spin‐coated P3HT films, and further in‐depth investigations identify that TrTPFB has nearly 100% polaron yielding efficiency and a high doping efficiency of over 80% in P3HT. These results suggest that the electrophilic‐attack doping mechanism has great potential for realizing highly efficient and universal doping in OSCs.

## 2 Results and Discussion

### 2.1 Universal p‐Doping Effect of TrTPFB

**Figure 1:**
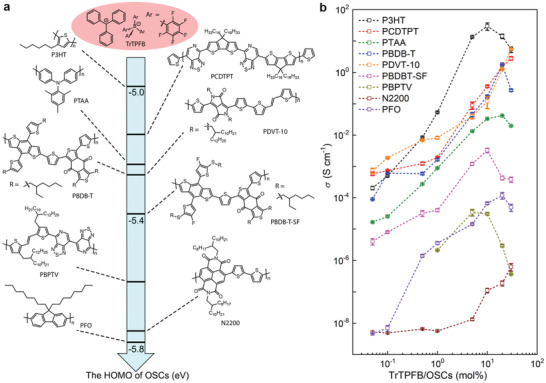
The universal p‐doping effect of TrTPFB. a) The molecule structure of TrTPFB and OSCs used in this study, with their HOMO levels illustrated. b) The electrical conductivity of a series of OSCs doped with TrTPFB as a function of doping concentration.

**Figure** **1**a shows the molecular structures of TrTPFB and the polymer semiconductors we have used as host semiconductors (the full names of the semiconductors are shown in Experimental Section). The conductivity of all semiconductor films is observed to increase by solution‐doping with TrTPFB (i.e., mixing the dopant with the OSCs in solution) regardless of the molecule structure or HOMO levels (Figure 1a). Specifically, the conductivity of P3HT at 10 mol% of TrTPFB doping concentration (corresponding to 10 dopants per 100 repeat units of 3‐hexylthiophene) was improved by more than 6 orders of magnitude compared with the pristine P3HT film. The conductivity of several other semiconductors such as PCDTPT, PBDB‐T, PTAA, and PDVT‐10, was improved by about 4 orders of magnitude; the conductivity of PBDB‐T‐SF and PBPTV was improved by about 3 orders of magnitude after doping. It is remarkable that the conductivity was enhanced by more than two orders of magnitude for N2200 and PFO with 30 mol% of TrTPFB, albeit their HOMO levels are around −5.8 eV. In fact, a p‐channel field‐effect transistor device based on N2200 can be realized by doping with TrTPFB, which clearly shows the p‐doping effect (see Section S1.3, Supporting Information). Such universal p‐doping effect on various OSCs regardless of the molecule structures and energy levels has rarely been observed in previous studies.

### 2.2 Doping Mechanism of TrTPFB

**Figure 2:**
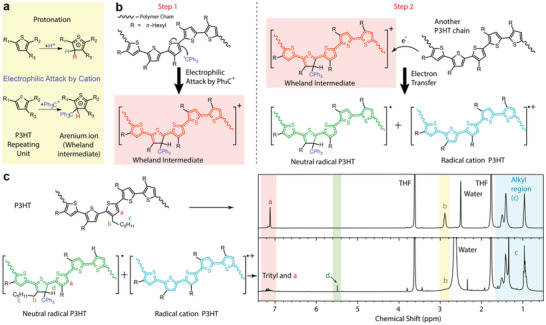
Schematic illustration and evidence for the electrophilic‐attack p‐doping mechanism. a) Schematic illustration of the protonation of thiophene involved in the protonation doping mechanism, and of the electrophilic‐attack‐based tritylation reaction that yields stabilized Wheland intermediate. Notably, although here we only show thiophene unit as an example, other conjugated systems such as benzene, thiazole, etc., can also be the targets for protonation or electrophilic attack. b) Proposed p‐doping mechanism of TrTPFB on P3HT in steps. The electrophilic attack of trityl cation on the thiophene unit yields thiophenium ion (the Wheland intermediate), which subsequently oxidizes another thiophene unit to achieve p‐doping. c) ^1^H NMR characterization of P3HT and TrTPFB‐doped P3HT. Successful tritylation (as suggested by the peak of the proton colored in green) and formation of radicals are clearly observed.

The universal p‐doping effect of TrTPFB shown above is undoubtedly an attractive property for dopants, and inspires us to understand the doping mechanism. Indeed, the LUMO and HOMO of TrTPFB were estimated to be −4.61 and −6.98 eV, respectively, by performing cyclic voltammetry (CV) and UV–vis–NIR measurements (Section S3, Supporting Information). Such energy levels of TrTPFB make it thermodynamically difficult to p‐dope P3HT through direct redox reaction doping, thus impelling us to propose a different doping mechanism for explaining the universal doping effect. Enlightened by the protonation‐doping mechanism (**Figure** **2**a, top), we propose the doping scheme using TrTPFB as the dopant for P3HT. Essentially, TrTPFB is a salt with a sterically hindered carbenium ion, i.e., the trityl cation Ph_3_C^+^. Carbenium ions are strong electrophiles as they are commonly seen in one of the earliest name reactions: Friedel–Crafts reaction.^[^ ^15^ ^]^ Thus, it is not surprising to see electrophilic attack of the trityl cation on thiophene, which is an electron‐rich arene. Importantly, this electrophilic attack by Ph_3_C^+^ would not lead to the Friedel–Crafts reaction in P3HT but result in the tritylated arenium (thiophenium) ion, alternatively called the Wheland intermediate (Figure 2a, bottom), which is structurally similar to the protonated thiophene but more stable because of the sterically hindered substituent (Ph_3_C) (see more details Section S5, Supporting Information).^[^ ^16^ ^]^ The existence of this Wheland intermediate can induce electron transfer from the neighboring P3HT chain that is responsible for p‐doping, resulting in two radical species: a neutral radical and a radical cation, as illustrated in Figure 2b.^[^ ^11^, ^17^ ^]^

To verify if the doping proceeds in the way we postulated, we employed ^1^H nuclear magnetic resonance (NMR) to characterize the precipitates which were produced when P3HT was doped with TrTPFB at a high doping ratio. These precipitates were supposed to contain no pristine P3HT or TrTPFB as they were purified carefully, and then dissolved in heated deuterated tetrahydrofuran (THF‐*d*_8_) for ^1^H NMR characterizations (see Section S4.1, Supporting Information). Interestingly, the peaks for trityl groups were seen, indicating the chemical bonding of the trityl group to P3HT chains. Additionally, a new peak at 5.5 ppm was observed after doping (peak d in Figure 2c), which can be assigned to the non‐aromatic proton on the thiophene unit formed upon electrophilic attack of trityl cation.^[^ ^11c^ ^]^ Moreover, ^1^H NMR results from a model study using a thiophene tetramer (OT_4_) further support our hypothesis on the covalent linking of the trityl groups to thiophene units through electrophilic attack and the assignment of peak d. In addition, the high‐resolution electrospray ionization‐mass spectrometry (ESI‐MS) characterizations on OT_4_ provide unambiguous evidence that the trityl group is chemically connected to the thiophene unit, forming the tritylated thiophenium (see Sections S4.2 and S4.3, Supporting Information).

Furthermore, all signals for the protons near the large conjugated system almost disappeared (peak b in Figure 2c), indicating the formation of radicals that move along the connected *π* bonds, as radicals can significantly shorten *T*_2_ relaxation time of nearby nuclei and make their peaks highly broadened and disappearing.^[^ ^18^ ^]^ However, it should be noted that the radical signals confirm the existence of radical cations but not assure the presence of the neutral radical species. In fact, previous studies highlighted that such neutral radicals were unstable against further chemical reactions and they reacted to release H_2_,^[^ ^17^ ^]^ which might be the case here but requires further investigations. Nevertheless, the above‐shown results confirm the validity of p‐doping by electrophilic attack of trityl cations on thiophenes. It is noticeable that as a strong electrophile, trityl cation may also induce hydride transfer from applicable positions. In the P3HT case, the pedant hexyl groups are too inert to serve as good hydride donors, and the NMR observations do not support the hydride transfer process. However, such H^−^ fetching behavior may become a factor that requires attention in other OSCs, such as the ones with more active benzylic methylene moieties.

The above‐shown electrophilic‐attack doping mechanism owns two prominent advantages: first, since all OSCs contain conjugated *π*‐systems, most of them can be the targets of an electrophilic attack as long as they are not sterically hindered. Thus, p‐doping through this electrophilic‐attack mechanism is likely to work on a wide range of OSCs regardless of their energetic levels, which is responsible for the universal doping effect shown in Figure 1b. Second, in contrast to the arenium ion resulted from the protonation of thiophene, which is usually an unstable species subject to fast re‐aromatization through deprotonation, the arenium ion formed through the tritylation of thiophene by a bulky trityl cation reported here has assured stability. In specific, by forcing the arenium ring to adopt a conformation unfavorable for the dissociation of proton (C—H bond at equatorial position, see Sections S5.1 and S5.2, Supporting Information), the deprotonation and re‐aromatization of the tritylated thiophenium is kinetically unfavorable;^[^ ^16^ ^]^ the stabilized arenium system thus promises the achievement of high doping efficiency, which will be further illustrated below. More importantly, this doping mechanism is not only applicable to trityl cation, but also to other strong electrophiles, such as nitronium tetrafluoroborate and diphenyliodium tetrakis(pentafluorophenyl)borate, which were also observed to produce significant p‐doping effect when they were mixed with P3HT (see Section S5.3, Supporting Information). Presumably, these compounds also undergo electrophilic attack on thiophene, generating arenium ions responsible for effective doping. Therefore, the electrophilic‐attack doping mechanism reported here not only ensures universal and efficient p‐doping of OSCs, but also is a general p‐doping mechanism that can be extended to numerous dopants.

### 2.3 Exceptionally High Doping Efficiency of TrTPFB

**Figure 3:**
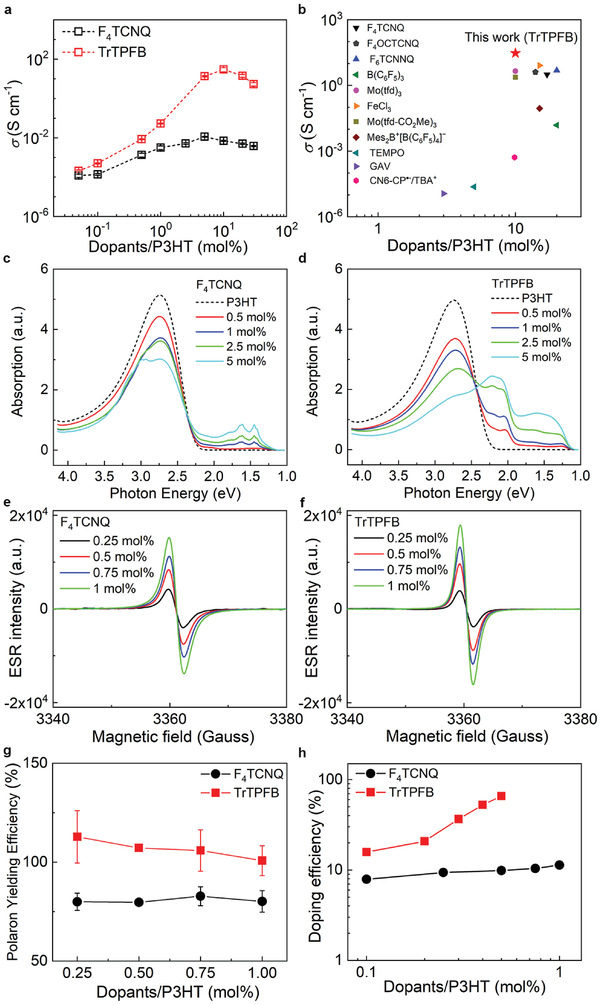
Investigations of the doping efficiency of TrTPFB. a) The electrical conductivity of P3HT doped with TrTPFB and F_4_TCNQ as a function of doping concentration. b) The conductivity of spin‐coated P3HT films doped by TrTPFB and other dopants from references^[^ ^8^, ^13^, ^22^ ^]^ using the solution‐doping method.^[^ ^22^, ^23^ ^]^ Optical absorption spectra of doped P3HT solutions with c) F_4_TCNQ as dopant and d) TrTPFB as dopant. ESR signals of e) F_4_TCNQ‐ and f) TrTPFB‐doped P3HT films at different doping ratios. g) The extracted polaron yielding efficiency for the two dopants. h) The doping efficiency extracted from the Mott–Schottky characterizations.

In this part, we performed quantitative studies to investigate the doping performance of TrTPFB to gain deeper insights into the superiority of the electrophilic‐attack doping mechanism. The strong doping capability of TrTPFB can be apparently seen from the considerably higher conductivity of TrTPFB‐doped P3HT than that of P3HT doped by a reference dopant F_4_TCNQ (**Figure** **3**a). The maximum conductivity of the P3HT film reaches 30 S cm^−1^ at 10 mol% of TrTPFB doping concentration, which is indeed among the highest conductivity values for spin‐coated P3HT films processed by solution‐doping method, as shown in Figure 3b. In fact, the superior doping performance of TrTPFB to F_4_TCNQ is not only observed in P3HT, but also in other polymer semiconductors (see Section S1.4, Supporting Information).

We then employed optical absorption spectroscopy to characterize the dopant‐induced polarons.^[^ ^6^, ^19^ ^]^ Since F_4_TCNQ has been well studied as a dopant for P3HT, it was taken as a reference dopant for the study.^[^ ^6^, ^20^ ^]^ The absorption spectra of F_4_TCNQ‐ and TrTPFB‐doped P3HT solutions as a function of doping ratio are shown in Figure 3c,d. A broad absorption in the range of 1.1–1.75 eV is observed upon doping in both solutions, which is ascribed to the dopant‐induced polarons on the P3HT backbone.^[^ ^19^, ^20^ ^]^ Aside from these absorptions, there also exists a broad absorption peak around 2.2 eV, which was assigned as the absorption of neutral ordered P3HT aggregates.^[^ ^20^, ^21^ ^]^ Previous studies show that the absorption peaks of P3HT polarons are located at about 1.4 and 1.6 eV,^[^ ^12^, ^13^, ^19^, ^20^ ^]^ based on which we can compare the doping magnitude of the two dopants by comparing their polaron absorption intensity. It is seen that the polaron absorption for TrTPFB at 1.6 eV is about 1.5 times higher than that for F_4_TCNQ at the same doping ratio, implying that more polarons are produced in TrTPFB‐doped P3HT (see Section S6.1, Supporting Information).

We further employed electron spin resonance (ESR) technique to characterize the polaron numbers and to estimate the polaron yielding efficiency, which is defined as the ratio between the number of polarons and the number of dopants, in the two doped films. The number of polarons at different doping concentrations can be quantitatively extracted from the ESR signals (Figure 3g and more details in Section S6.2, Supporting Information). It is found that the polaron yielding efficiency of F_4_TCNQ is about 80% in the doping range of 0.25–1 mol%, which is consistent with previous results.^[^ ^6^, ^12^, ^20^ ^]^ In comparison, the polaron yielding efficiency of TrTPFB is as high as 100%, indicating that each dopant (trityl cation) can induce a polaron in P3HT, which is observed for the first time in doped P3HT.

However, it is notable that the production of polarons is only the first key step in the doping process, which results in ion pairs (polaron and dopant anion) that are bound by Coulombic force. Following that ion pairs need to overcome Coulombic binding by thermally dissociating into separate charge carriers.^[^ ^24^ ^]^ Consequently, the doping efficiency, which is defined as the ratio of number of free charge carriers to number of dopant molecules, is determined by the efficiency of the two processes and is generally lower than the polaron yielding efficiency. To estimate the doping efficiency of the two dopants, Mott–Schottky analysis was performed on metal–insulator–semiconductor (MIS) diodes for extraction of dopant‐induced charge carrier density^[^ ^25^ ^]^ (see Section S6.3, Supporting Information). The doping efficiency of the two dopants is shown in Figure 3h. F_4_TCNQ was found to have doping efficiency of about 10% in the doping range of 0.1–1 mol%, which is consistent with previous reports.^[^ ^12^ ^]^ In contrast, the doping efficiency of TrTPFB is around 20% at low doping concentration, possibly due to the trap‐filling of charge carriers, while this value quickly increases to 80% at the doping ratio of 0.5 mol%, which, to the best of our knowledge, is among the highest doping efficiency values ever reported for solution‐doped P3HT.^[^ ^7^ ^]^ In particular, due to the super linear increase of charge carrier density with doping ratios as seen in Figure 3h, it is very likely that the doping efficiency of TrTPFB for P3HT can be further improved as doping ratios increases. Such results unambiguously demonstrate the excellent doping ability of TrTPFB as a p‐dopant.

In view of the high doping performance of TrTFFB on P3HT, we evaluated the thermoelectric performance of this system by measuring the Seebeck coefficients (*S*) of doped P3HT films. The power factor (*PF*), which is an important figure of merit for characterizing thermoelectric performance, was obtained by equation: *PF* = *S* ^2^ *σ*. The maximum *PF* of P3HT films doped by TrTPFB reaches 27.8 µW m^−1^ K^−2^. This *PF* value is also among the highest ones for spin‐coated P3HT films processed by solutiondoping method (see Section S7, Supporting Information). All these results directly illustrate the outstanding doping ability of TrTPFB.

### 2.4 The Influence of Anion Structure on Doing Performance

**Figure 4:**
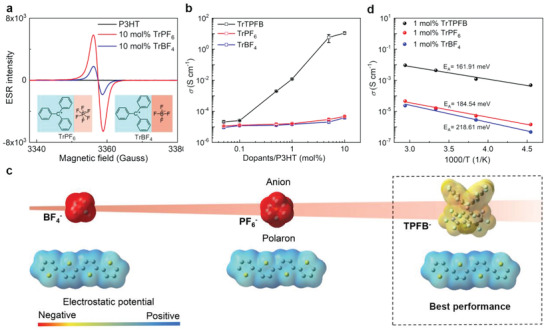
Influence of anion structure on doping performance. (a) The ESR spectra of P3HT films doped with TrBF_4_ and TrPF_6_. The molecular structures of two organic salts are shown in the inset. (b) The conductivity of doped P3HT as a function of doping concentration for the three dopants. (c) Schematic diagram showing the dependence of polaron‐anion Coulombic bounding on the anion sizes. The electrostatic surface potential (ESP) of all cations and anions is determined by density of functional theory (DFT) calculations. (d) The temperature‐dependent conductivity of P3HT doped with the three dopants.

Having identified the doping mechanism of trityl cation and its doping performance, it is intriguing to know how the [B(C_6_F_5_)_4_]^−^ anion (TPFB^−^) affects the doping process. Thus, we have carried out preliminary investigations by characterizing the doping performance of two other organic salts consisting of trityl cation but different anions: trityl tetrafluoroborate (TrBF_4_) and trityl hexafluophosphate (TrPF_6_). **Figure** **4**a shows the ESR spectra of P3HT films doped by the two organic salts, which apparently illustrates their doping effect. Meanwhile, it is noted that the conductivity of P3HT doped by these two dopants is much lower compared to the one doped by TrTPFB at the same dopant concentration (see Figure 4b), implying the weaker doping performance of these two dopants.

These results suggest the molecule structure of the anion definitely affects the doping efficiency and thus should be properly chosen. Our results show that [B(C_6_F_5_)_4_]^−^ is preferred over BF_4_^−^ and PF_6_^−^ for achieving higher doping efficiency. This is probably accounted by several reasons: (1) the delocalization of negative charge over a large anion such as [B(C_6_F_5_)_4_]^−^ helps with the stabilization of the polaron and more importantly, could be favorable for the electrophilic attack reaction shown above, as revealed by Beljonne et al. through theoretical calculations,^[^ ^17^ ^]^ and (2) the bulky structure of [B(C_6_F_5_)_4_]^−^ leads to low negative electrostatic surface potential and reduces the electrostatic attractive force between it and the polaron, which is beneficial to obtain efficient ion pair dissociation or higher charge dissociation efficiency^[^ ^22^, ^26^ ^]^ (see Figure 4c). Indeed, the doped P3HT films were seen to exhibit increasing activation energy with the decrease of the anion size according to temperature‐dependent conductivity measurements (Figure 4d), which is partly attributed to the increasing electrostatic binding energies between the generated polarons and the anions. Besides the ability to offer high doping efficiency, the [B(C_6_F_5_)_4_]^−^ was also reported to be an inert non‐coordinating counterion that is moisture‐ and air‐resistant, which is important to stable doping.^[^ ^13c^ ^]^ The results shown in Section S8 (Supporting Information) demonstrate the high storage stability of TrTPFB‐doped P3HT films.

## 3 Conclusion

To summarize, we reveal a new p‐doping mechanism relying on the electrophilic attack of cations on OSCs by investigating the interaction between TrTPFB and P3HT. This doping mechanism intrinsically possesses significant advantages over the previously reported doping mechanisms, and endows the corresponding dopants with ability to universally and efficiently p‐dope OSCs. We show that TrTPFB exhibits remarkable doping performance with the ability to dope various OSCs, and to enhance the conductivity of P3HT to 30 S cm^−1^. In‐depth investigations indicate the polaron yielding efficiency and doping efficiency of TrTPFB reach ultrahigh values of 100% and 80%, respectively, in P3HT. Besides, we find that although the anion is not directly involved in the doping reaction, its structure greatly affects the doping efficiency and thus should be properly chosen. Our study casts new light on the doping mechanisms of OSCs, and provides a route to realize efficient doping of various OSCs.

## 4 Experimental Section

### Materials

Poly[4‐(4,4‐dihexadecyl‐4H‐cyclopenta[1,2‐b:5,4‐b']‐dithiophen‐2‐yl)‐alt‐[1,2,5]thiadiazolo‐[3,4‐c]pyridine] (PCDTPT), Poly[[4,8‐bis[5‐(2‐ethylhexyl)‐2‐thienyl]benzo[1,2‐b:4,5‐b']dithiophene‐2,6‐diyl]‐2,5‐thiophenediyl[5,7‐bis(2‐ethylhexyl)‐4,8‐dioxo‐4H,8H‐benzo[1,2‐c:4,5‐c'] dithiophene‐1,3‐diyl]] (PBDB‐T), Poly[bis(4‐phenyl)(2,4,6‐trimethylphenyl)amine (PTAA), Poly{3,6‐dithiophen‐2‐yl‐2,5‐di(2‐decyltetradecyl)‐pyrrolo[3,4‐c]pyrrole‐1,4‐dione‐alt‐thienylenevinylene‐2,5‐yl} (PDVT‐10), Poly[(2,6‐(4,8‐bis(5‐(2‐ethylhexylthio)‐4‐fluorothiophen‐2‐yl)‐benzo[1,2‐b:4,5‐b’]dithiophene))‐alt‐(5,5‐(1’,3’‐di‐2‐thienyl‐5’,7’‐bis(2‐ethylhexyl)benzo[1’,2’‐c:4’,5’‐c’]dithiophene‐4,8‐dione)] (PBDB‐T‐SF), Pyridal[2,1,3]thiadiazole‐based semiconducting polymer (PBPTV), Poly{[*N*,*N*'‐bis(2‐octyldodecyl)naphthalene‐1,4,5,8‐bis(dicarboximide)‐2,6‐diyl]‐alt‐5,5'‐(2,2'‐bithiophene)} (N2200) and Polydioctylfluorene (PFO) were purchased from commercial companies and used as received without further purifications. P3HT were purchased from Aladdin Reagent Co., Ltd.

### Preparation of Doped Solutions and Films

TrTPFB (from Strem Chemicals, Inc.), F_4_TCNQ, TrPF_6_, TrBF_4_ (from TCI (Shanghai) Development Co., Ltd) and all OSCs were dissolved in chlorobenzene to prepare semiconductor solutions with different dopant concentrations unless specified with other solvents. The concentration of P3HT solution was 20 g L^−1^, while the solution concentrations of TrTPFB (0.1, 0.5, 1, 5, and 10 g L^−1^), F_4_TCNQ (0.1 and 0.5 g L^−1^), TrPF_6_ (0.1 and 0.5 g L^−1^) and TrBF_4_ (0.1 and 0.5 g L^−1^) varied with the doping ratio. The blended solutions were stirred to be homogeneous prior to film preparation. All solutions were filtered through the 0.45 µm syringe filters to remove impurities or aggregates before usage. Semiconductor films were made through spin‐coating of solutions in glovebox, and different post‐annealing conditions were used for different semiconductors (Section S1, Supporting Information)

### CV, NMR, and MS Measurements

For Cyclic voltammogram (CV) of TrTPFB solution in dichloromethane (1.0 mm), redox potentials were determined by using 0.10 m n‐Bu_4_N^+^PF_6_^−^ as a supporting electrolyte, and the electrode potential was externally calibrated by the ferrocene/ferrocenium redox couple. ^1^H NMR and heteronuclear mutiple bond correlation (HMBC) spectra were recorded on a Bruker AVANCE Neo 400 spectrometer. Typically, the OT_4_ CDCl_3_ solutions and TrTPFB CDCl_3_ solutions were mixed at 100 mol% and stored in the glovebox for 24 h before ^1^H NMR and HMBC measurement at room temperature. The precipitate produced by TrTPFB doped P3HT was dissolved in THF‐*d*_8_ and recorded at 60 °C. High resolution ESI‐MS characterization were carried out with a Bruker compact quadrupole time‐of‐flight mass spectrometry system (QTOF‐MS). The doped OT_4_ solutions were dispersed in methanol and filtered before the measurement.

### UV–Vis–NIR and ESR Measurements

The ultraviolet–visible–near‐infrared (UV–vis–NIR) absorption spectra of solution samples were carried out with UV‐3600PLUS (SHIMADZU). For ESR measurements, the prepared sample solutions were dropped onto glass substrates and dried in an Ar‐filled glove box to remove the solvent, and then placed into paramagnetic tubes. After sealing the paramagnetic tubes, the ESR was measured on a JEOL JES‐FA200 ESR spectrometer at room temperature.

### Measurement of Electrical Conductivity

Patterned electrodes (Cr/Au: 2 nm/30 nm) were prepared on Si wafers with 300 nm SiO_2_ by photolithography. The doped semiconductor solutions were spin‐coated to form a thin film on the substrate at a speed of 1500 rpm for 30 s and annealed at the corresponding temperature and time (see Table S1, Supporting Information in detail). The conductivity of the prepared devices was measured in air by a four‐probe method through a Keithley 4200 semiconductor analyzer.

### Preparation and Measurement of OFETs and MIS Diodes

In this work, top‐gate, bottom‐contact OFETs were fabricated. First, patterned electrodes (Cr/Au: 2 nm/30 nm) were prepared on a glass substrate by photolithography. Second, the substrate was sonicated for 5 min in ultrapure water, acetone, and isopropanol in sequence. Third, the prepared dopant/OSC solutions were spin‐coated on the substrate at 1500 rpm for 30 s, and then they were annealed according to the corresponding conditions (see Table S1, Supporting Information). Subsequently, Cytop solution was spin‐coated at 1000 rpm for 30 s and baked at 90 °C for 20 min as a dielectric layer. Finally, Al with a thickness of about 100 nm was deposited as a gate electrode through a shadow mask. The OFETs were measured in an Ar‐filled glove box by an Angilent B2912A source meter.

MIS Diodes were prepared by spin‐coating the prepared doping solutions on ITO glass substrates with about 30 nm Al_2_O_3_ deposited by ALD Atomic Layer Deposition (ALD), following which about 40 nm of Au was evaporated. Next, the capacitance‐voltage measurement was performed on Agilent 4294A precision impedance analyzer at frequency of 100 Hz, AC voltage of 0.5 V, with DC bias voltage in the range of 10 to −10 V.

### Characterization of Thermoelectric Performance

To measure the Seebeck coefficient of the doped samples, a home‐made thermoelectric measurement setup was used. The devices containing one heater, two thermometers which also act as electrical contacts were fabricated by photolithographic patterning of metal bilayers of Cr (10 nm) and Au (15 nm) on glass substrates. To obtain Seebeck coefficient $S\;=\frac{\Delta V}{\Delta T}\;$, the temperature gradient between the two electrodes was estimated by converting the resistance of electrodes into temperature using the temperature‐coefficient‐of resistance (TCR) (see Section S7, Supporting Information), and the built‐in thermal voltage was measured using Keithley nanovoltmeter model 2182A. All Seebeck coefficients were measured at 300 K in high vacuum (<10^−5^ mbar) using Janis ST‐100.

### ESP Calculation

DFT calculations were performed with the Gaussian 09 program. All geometry optimizations were carried out at the B3LYP level of DFT with the 6–31G (d, p) basis set. ESP of five compounds were calculated at B3LYP/6‐31G (d, p) level.

## Conflict of Interest

The authors declare no conflict of interest.

## Author Contributions

J.G. performed the characterizations of electrical conductivity as well as the absorption spectra of different organic semiconductors doped by different dopants. Y.L., Y.Y., and Y.B. performed NMR and MS analyses. X.W., Y.W., J.G., and Z.Z. provided help with absorption spectra and ESR measurements. J.G., P.C., and X.Q. performed the fabrication and measurement of MIS diodes. P.C. performed measurement of the Seebeck Coefficient. H.C. provided polymer semiconductor materials. L.J. and L.L. provided help with NMR and electrical measurements. Y.B., T.‐Q.N., and Y.H. conceived the idea and supervised the project. J.G., Y.L., S.W., Y.B., T.‐Q.N., and Y.H. wrote the manuscript. All the authors revised and approved the manuscript.

## Supporting information

Supporting Information

## Data Availability

The data that support the findings of this study are available from the corresponding author upon reasonable request.
