# Supplementary material for: Revealing the Electrophilic‐Attack Doping Mechanism for Efficient and Universal p‐Doping of Organic Semiconductors
Source: Adv Sci (Weinh). 2022 Sep 11;9(32):2203111. doi: 10.1002/advs.202203111 (PMC9661849; doi:10.1002/advs.202203111)
Supplement: Supplementary file 1 — Supporting Information [file ADVS-9-2203111-s001.pdf]

ADVANCED SCIENCE
Open Access

Supporting Information

for *Adv. Sci.*, DOI 10.1002/advs.202203111

Revealing the Electrophilic-Attack Doping Mechanism for Efficient and Universal p-Doping of Organic Semiconductors

Jing Guo, Ying Liu, Ping-An Chen, Xinhao Wang, Yanpei Wang, Jing Guo, Xincan Qiu, Zebing Zeng, Lang Jiang, Yuanping Yi, Shun Watanabe, Lei Liao, Yugang Bai, Thuc-Quyen Nguyen* and Yuanyuan Hu**

Revealing the Electrophilic-Attack Doping Mechanism for Efficient and Universal p-doping of Organic Semiconductors

Jing Guo[1], *Ying Liu*[2], *Ping-An Chen*[1], *Xinhao Wang*[2], *Yanpei Wang*[2], *Jing Guo*[2], *Xincan Qiu*[1], *Zebing Zeng*[2], *Lang Jiang*[3], *Yuanping Yi*[3], *Shun Watanabe*[4], *Lei Liao*[1], *Yugang Bai*[2*], *Thuc-Quyen Nguyen*[5*], *Yuanyuan Hu*[1,6]*

[1]International Science and Technology Innovation Cooperation Base for Advanced Display Technologies of Hunan Province, College of Semiconductors (College of Integrated Circuits), Hunan University, Changsha 410082, China
[2]State Key Laboratory of Chem-/Bio-Sensing and Chemometrics, School of Chemistry and Chemical Engineering, Hunan University, Changsha, Hunan, 410082, China
[3]Beijing National Laboratory for Molecular Sciences, Key Laboratory of Organic Solids, Institute of Chemistry, Chinese Academy of Sciences, Beijing 100190, China
[4]Material Innovation Research Center (MIRC) and Department of Advanced Material Science, Graduate School of Frontier Sciences, The University of Tokyo, 5-1-5 Kashiwanoha, Kashiwa, Chiba, 77-8561, Japan
[5]Center for Polymers and Organic Solids, Department of Chemistry and Biochemistry, University of California at Santa Barbara, Santa Barbara, California 93106, United States
[6]Shenzhen Research Institute of Hunan University, Shenzhen 518063, China

Email of the corresponding authors:
baiyugang@hnu.edu.cn; quyen@chem.ucsb.edu; yhu@hnu.edu.cn

Table of Contents

S1. Doping of different OSCs with TrTPFB and F_4TCNQ 33

S1.1 Preparation of doped organic semiconductor films 33

S1.2 ESR spectra of doped-OSCs with TrTPFB.. 44

S1.3 Characterization of the performance of OFETs based on TrTPFB-doped OSCs 55

S1.4 Doping of various OSCs with F_4TCNQ 99

S2. Effect of dopants (TrTPFB and F_4TCNQ) on morphology of P3HT films. 1010

S3. Estimation of LUMO and HOMO levels of TrTPFB 1212

S4. Experimental evidence showing the electrophilic attack of trityl cations on thiophenes 1313

S4.1 NMR characterization.. 1313

S4.2 Characterization of doped OT_4 1414

S4.3 Further discussions on NMR characterization of doped OT_4 1716

S5. Further discussions on the doping mechanism of Brønsted acids, TrTPFB and other electrophiles.. 1817

S5.1. The role of Wheland intermediate in the acid doping process 1817

S5.2. Rational design for the stabilization of the Wheland intermediate......... 2019

S5.3. Extension of the doping mechanism to other electrophiles 2221

S6. Characterization of dopant polaron yielding efficiency and doping efficiency 2423

S6.1 Absorption spectra characterizations 2423

S6.2 ESR characterizations.. 2827

S6.3 Mott-Schottky analysis 2928

S7. Characterization of thermoelectric performance 3029

S8. Stability of TrTPFB-doped P3HT films... 3332

S1. Doping of different OSCs with TrTPFB and F_4TCNQ

S1.1 Preparation of doped organic semiconductor films

A series of OSCs were employed as host semiconductors for the doping studies in this work. The molecule structures and energy levels of these OSCs are shown in Figure 1. The OSC films were made by blending the OSC solutions with dopant solutions, and then spin-coated onto substrates. Here we show the post-annealing conditions for these OSC films, as listed in the Table below.

Table S1. Annealing temperature and thermal annealing time of thin film samples.

Host OSCs	P3HT	PCDTPT	PBDB-T	PTAA	PDVT-10	PBDB-T-SF	PBPTV	N2200	PFO
Annealing temperature (°C)	**130**	**200**	**200**	**150**	**180**	**200**	**180**	**150**	**150**
Annealing time (min)	**5**	**8**	**10**	**10**	**5**	**10**	**5**	**20**	**10**

S1.2 ESR spectra of doped-OSCs with TrTPFB

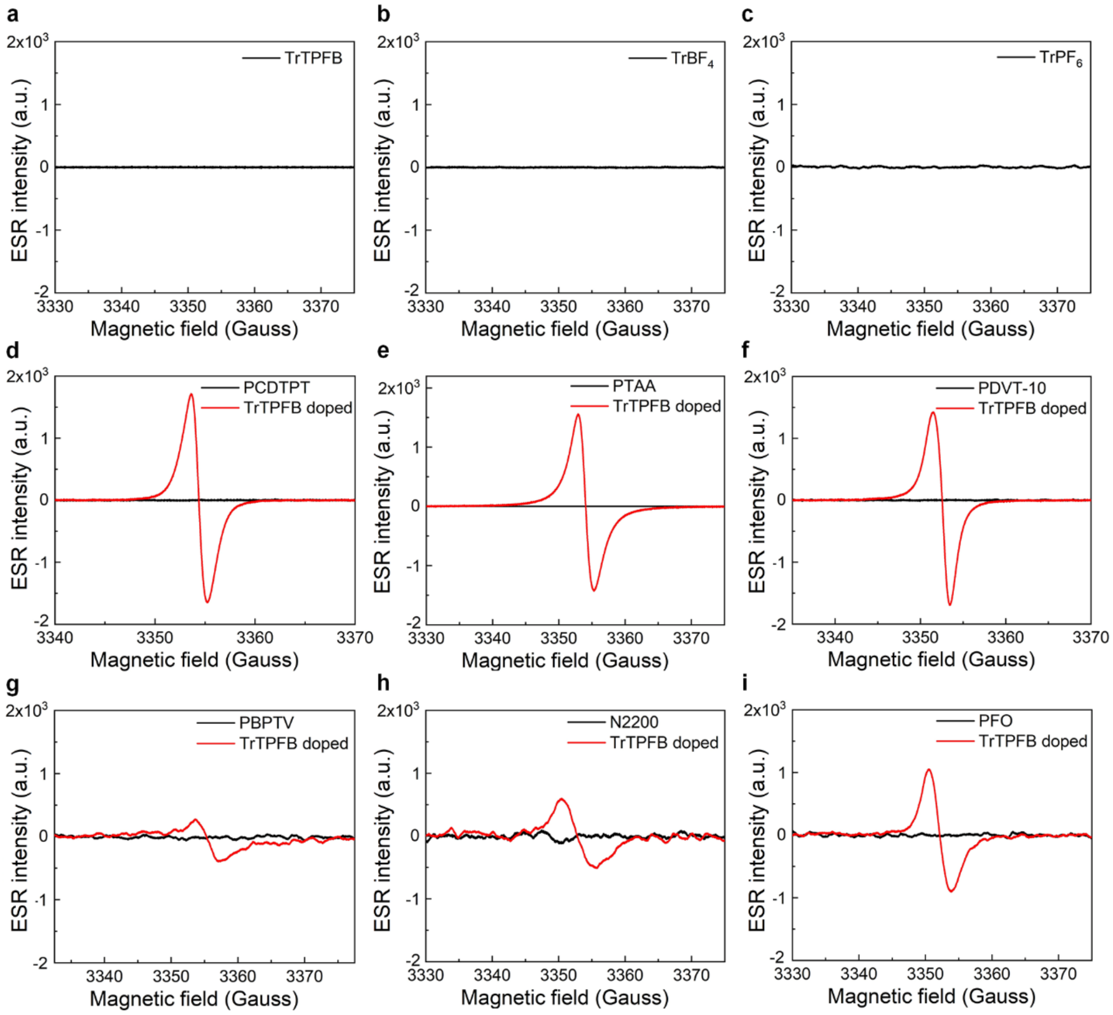

Figure S1. The ESR spectra of three dopants, pristine OSCs and TrTPFB doped OSC films. (a) TrTPFB, (b) $TrBF_4$, (c) $TrPF_6$, pristine and TrTPFB-doped (d) PCDTPT, (e) PTAA, (f) PDVT-10, (g) PBPTV, (h) N2200 and (i) PFO.

S1.3 Characterization of the performance of OFETs based on TrTPFB-doped OSCs

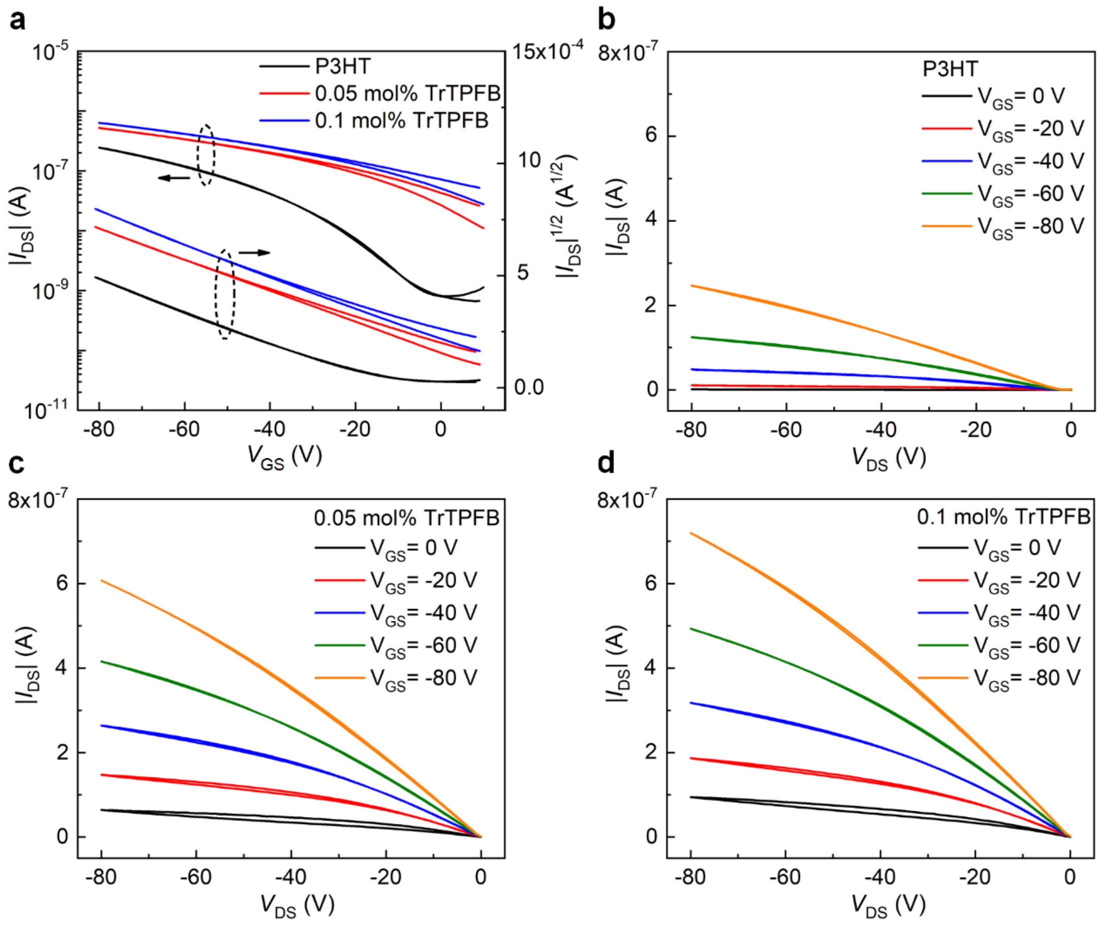

Figure S2. The electrical properties of TrTPFB-doped P3HT OFETs (TGBC structure device) with different doping concentrations. (a) The transfer characteristics of pristine, 0.05 mol% and 0.1 mol% TrTPFB doped P3HT OFETs. The output characteristics of (b) pristine, (c) 0.05 mol% and (d) 0.1 mol% TrTPFB-doped P3HT OFETs.

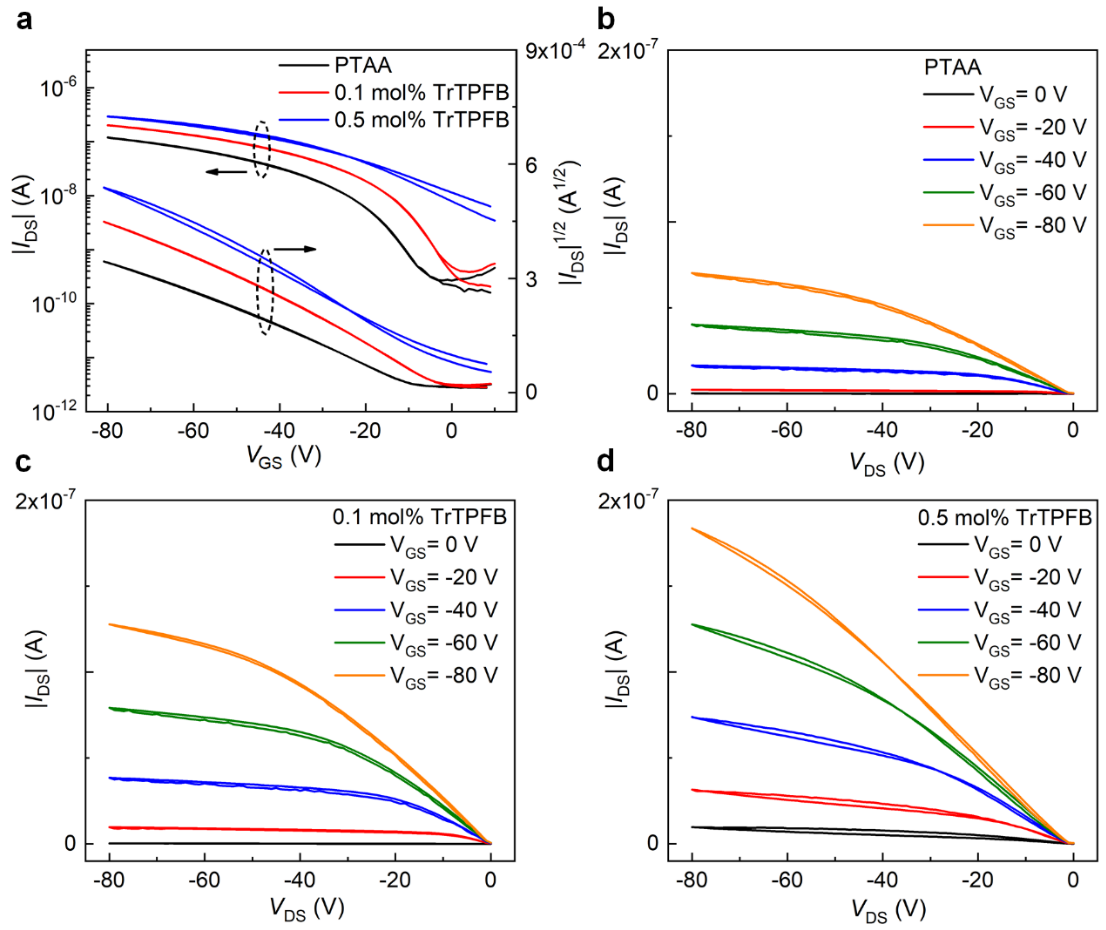

Figure S3. The electrical properties of TrTPFB-doped PTAA OFETs (TGBC structure device) with different doping concentrations. (a) The transfer characteristics of pristine, 0.1 mol% and 0.5 mol% TrTPFB doped PTAA OFETs. The output characteristics of (b) pristine, (c) 0.1 mol% TrTPFB and (d) 0.5 mol% TrTPFB-doped PTAA OFETs.

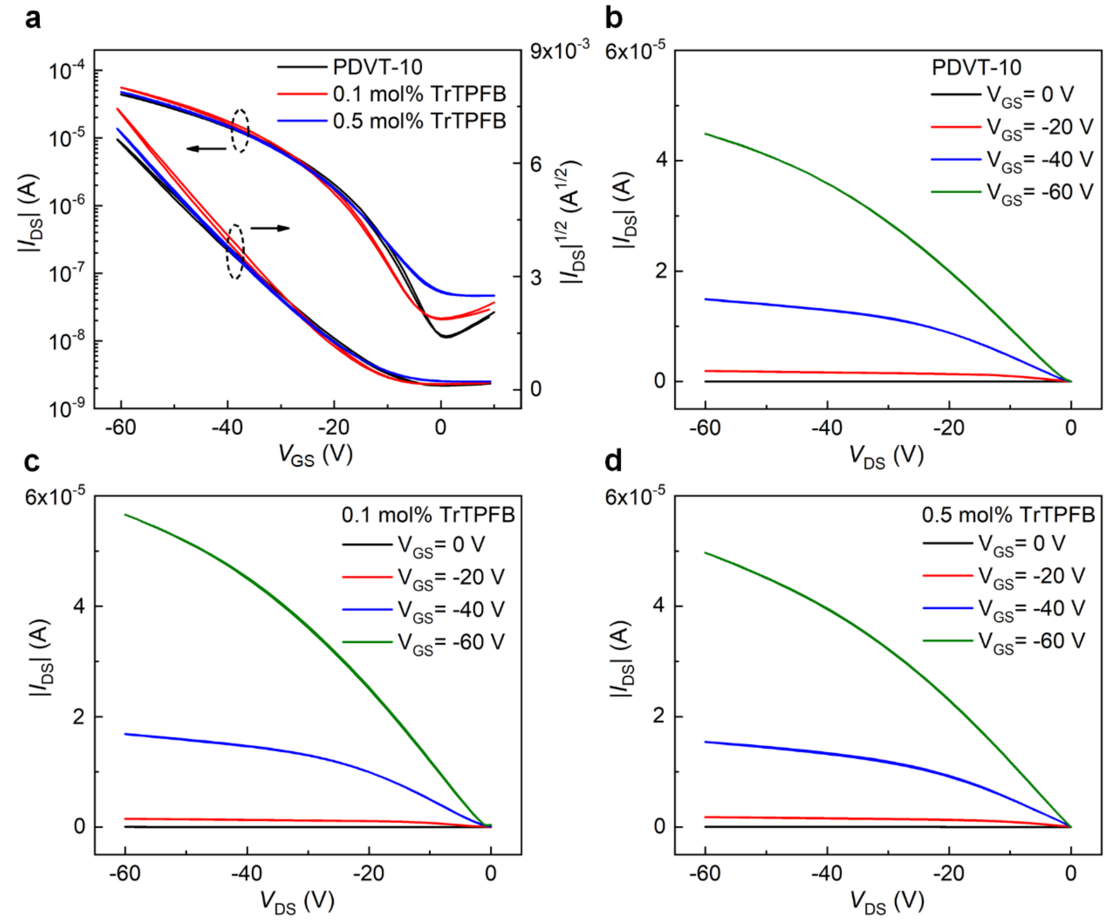

Figure S4. The electrical properties of TrTPFB-doped PDVT-10 OFETs (TGBC structure device) with different doping concentrations. (a) The transfer characteristics of pristine, 0.1 mol% and 0.5 mol% TrTPFB doped PDVT-10 OFETs. The output characteristics of (b) pristine, (c) 0.1 mol%, (d) 0.5 mol% TrTPFB-doped PDVT-10 OFETs.

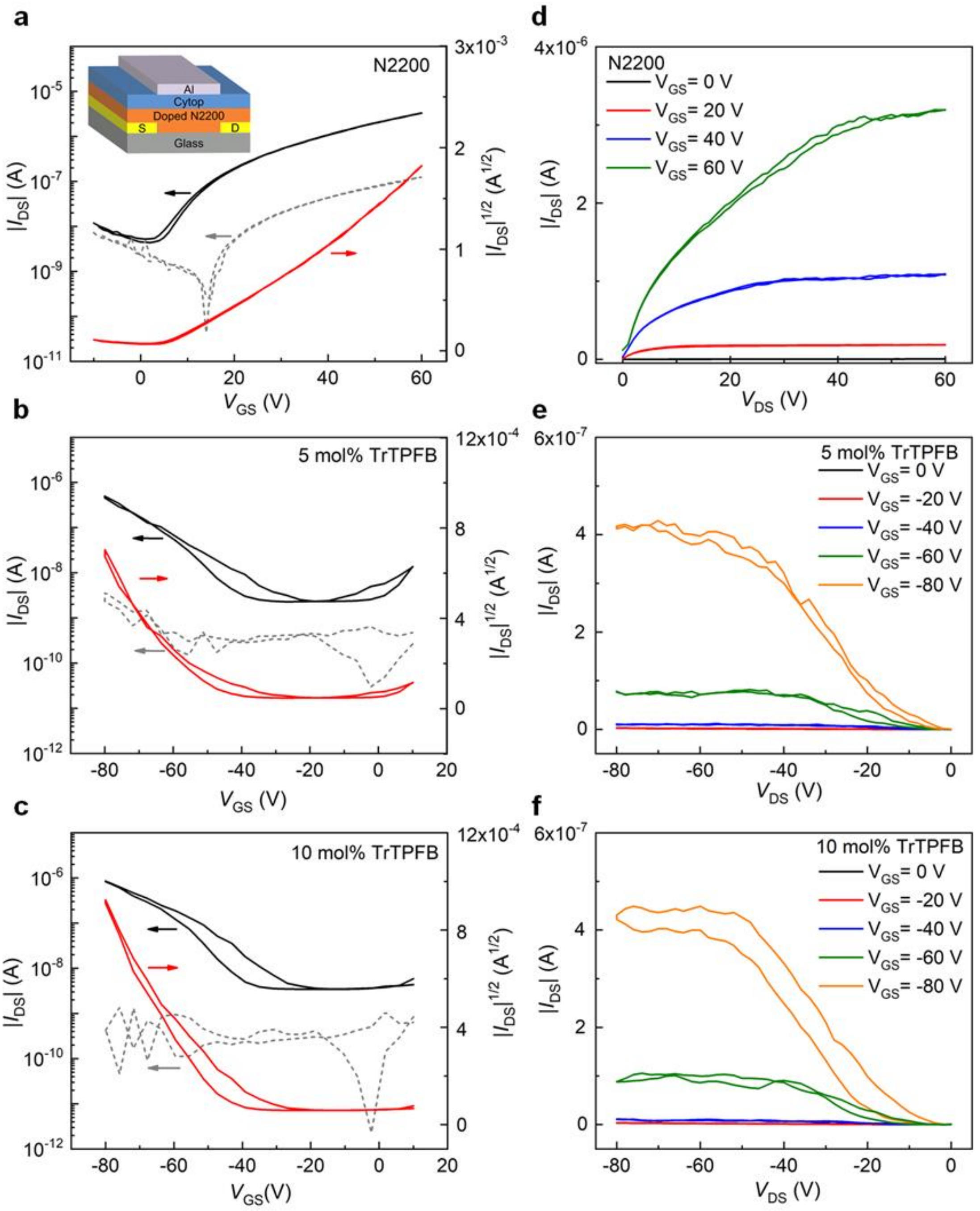

Figure S5. The electrical properties of TrTPFB-doped N2200 OFETs (TGBC structure device) with different doping concentrations. The transfer characteristics of (a) pristine and TrTPFB-doped N2200 OFETs with doping ratio of (b) 5 mol% and (c) 10 mol%. The output characteristics of (d) pristine and TrTPFB-doped N2200 OFETs with doping ratio of (e) 5 mol% and (f) 10 mol%. (The inset is a schematic diagram of devices with TGBC structure.)

S1.4 Doping of various OSCs with F_4TCNQ

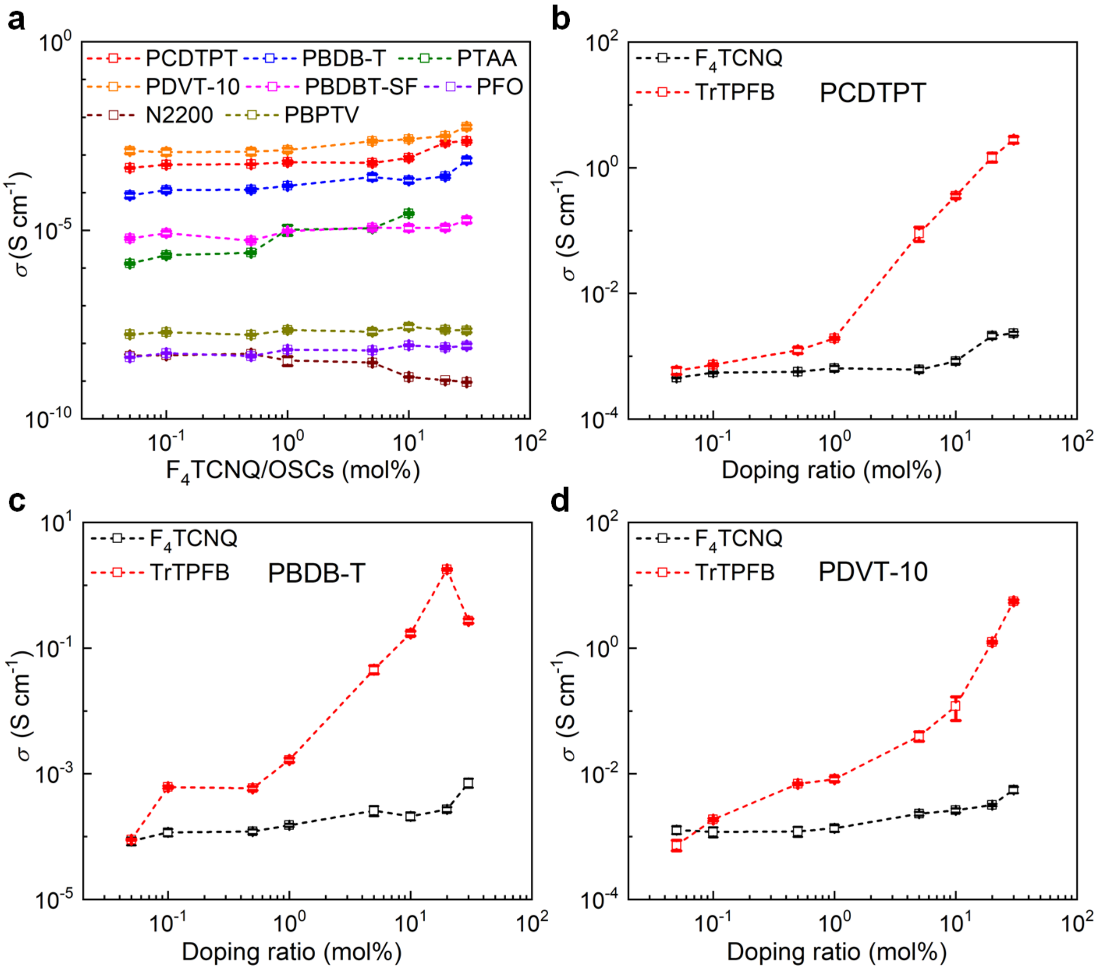

Figure S6. Comparison of the electrical conductivity of different semiconductors doped with F_4TCNQ and TrTPFB. (a) The conductivity of a series of organic semiconductors doped with different concentrations of F_4TCNQ. Comparison of the conductivity of F_4TCNQ and TrTPFB doped (b) PCDTPT, (c) PBDB-T and (d) PDVT-10. The results show that only those with HOMO no deeper than −5.3 eV can be doped according to the conductivity measurements

S2. Effect of dopants (TrTPFB and F_4TCNQ) on morphology of P3HT films

In the manuscript, we did not stress the good solubility of TrTPFB compared to F_4TCNQ or other dopants, but this indeed is also an important property for dopants. Especially, TrTPFB causes less aggregation effect in doped P3HT, which can be seen from the atomic force microscopy (AFM) and optical microscopy images shown below.

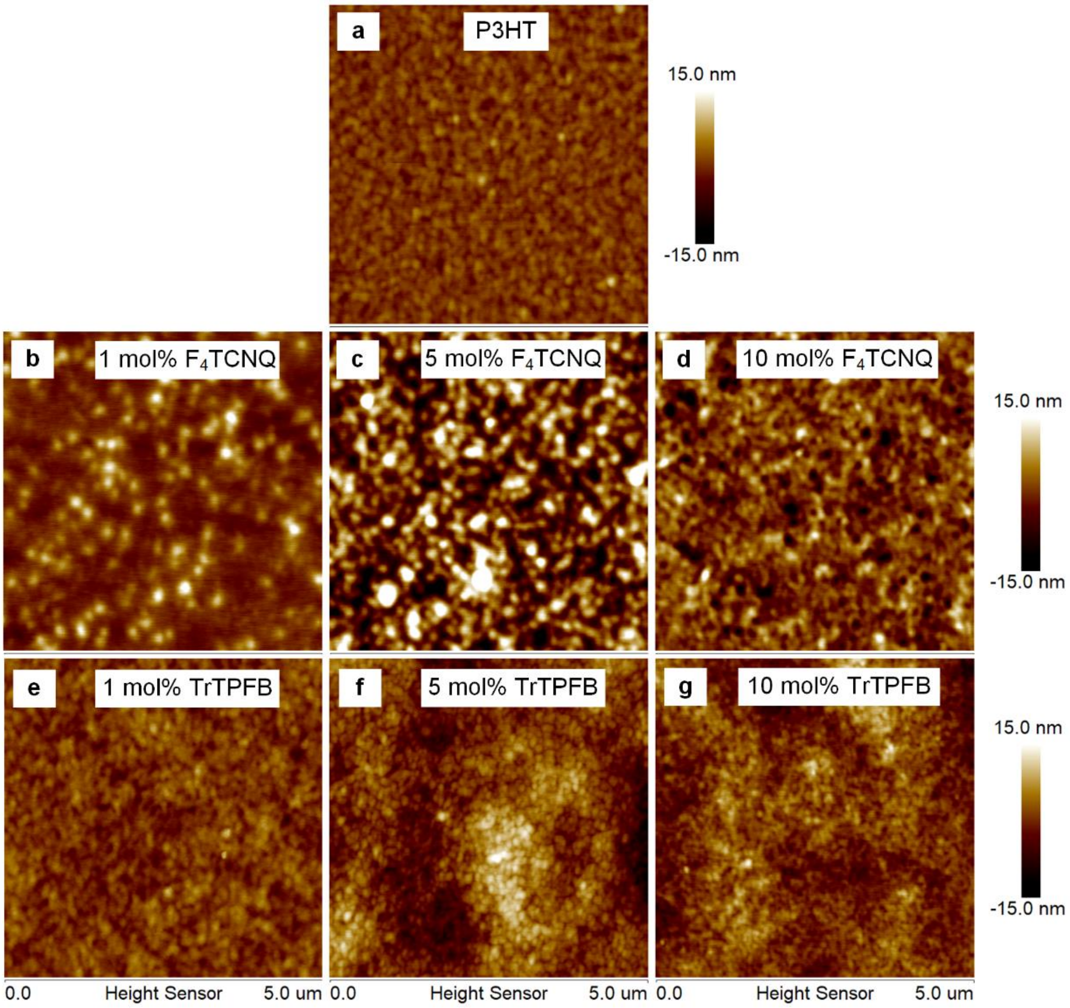

Figure S7. The topography (5×5 μm) of pristine, TrTPFB and F_4TCNQ-doped P3HT films obtained by AFM measurement. (a) Pristine P3HT, (b-d) 1 mol%, 5 mol% and 10 mol% F_4TCNQ -doped films, respectively, and (e-g) 1 mol%, 5 mol% and 10 mol% TrTPFB-doped films, respectively.

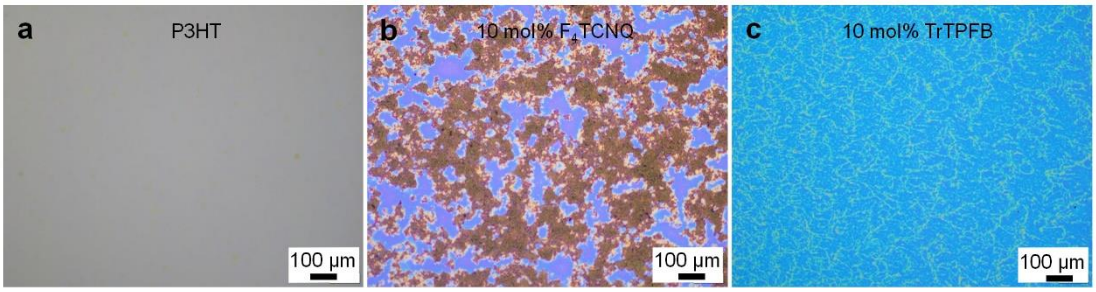

Figure S8. The surface topography of pristine and dope P3HT films obtained by optical microscope. (a) Pristine P3HT, (b) 10 mol% F_4TCNQ and (c) 10 mol% TrTPFB doped P3HT films prepared by spin-coating.

At the doping ratio of 10 mol%, the TrTPFB-doped P3HT film looks continuous even though it is not that smooth. However, the F_4TCNQ-doped P3HT film exhibit discontinuous features with strong aggregation effects.

S3. Estimation of LUMO and HOMO levels of TrTPFB

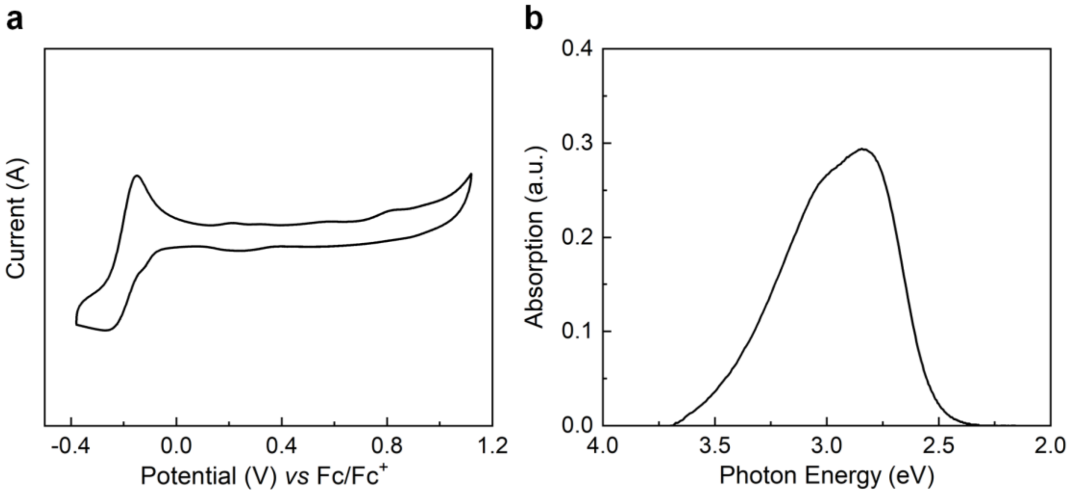

Figure S9. Estimation of the LUMO and HOMO levels of TrTPFB. (a) The cyclic voltammetry of TrTPFB in dichloromethane (0.10 M n-$Bu_4N^+PF_6^-$ as a supporting electrolyte). (b) The UV-vis-NIR absorption spectrum of TrTPFB in chlorobenzene solution. The cyclic voltammetry measurement shows TrTPFB has only one quasi-reversible reduction wave, and its reduction potential is $V_{red} - V_{FC}/V_{FC}^{+} = -0.19\,eV$. The optical energy gap of TrTPFB is $E_g^{opt} = 2.37\,eV$, which is obtained through UV-vis-NIR absorption spectra. The energy levels of LUMO and HOMO are then estimated to be −4.61 eV and −6.98 eV, respectively, which are determined by the formulas of $LUMO = -(4.8 + (V_{red} - V_{FC}/V_{FC}^{+}))$ and $HOMO = LUMO - E_g^{opt}$.

S4. Experimental evidence showing the electrophilic attack of trityl cations on thiophenes

S4.1 NMR characterization

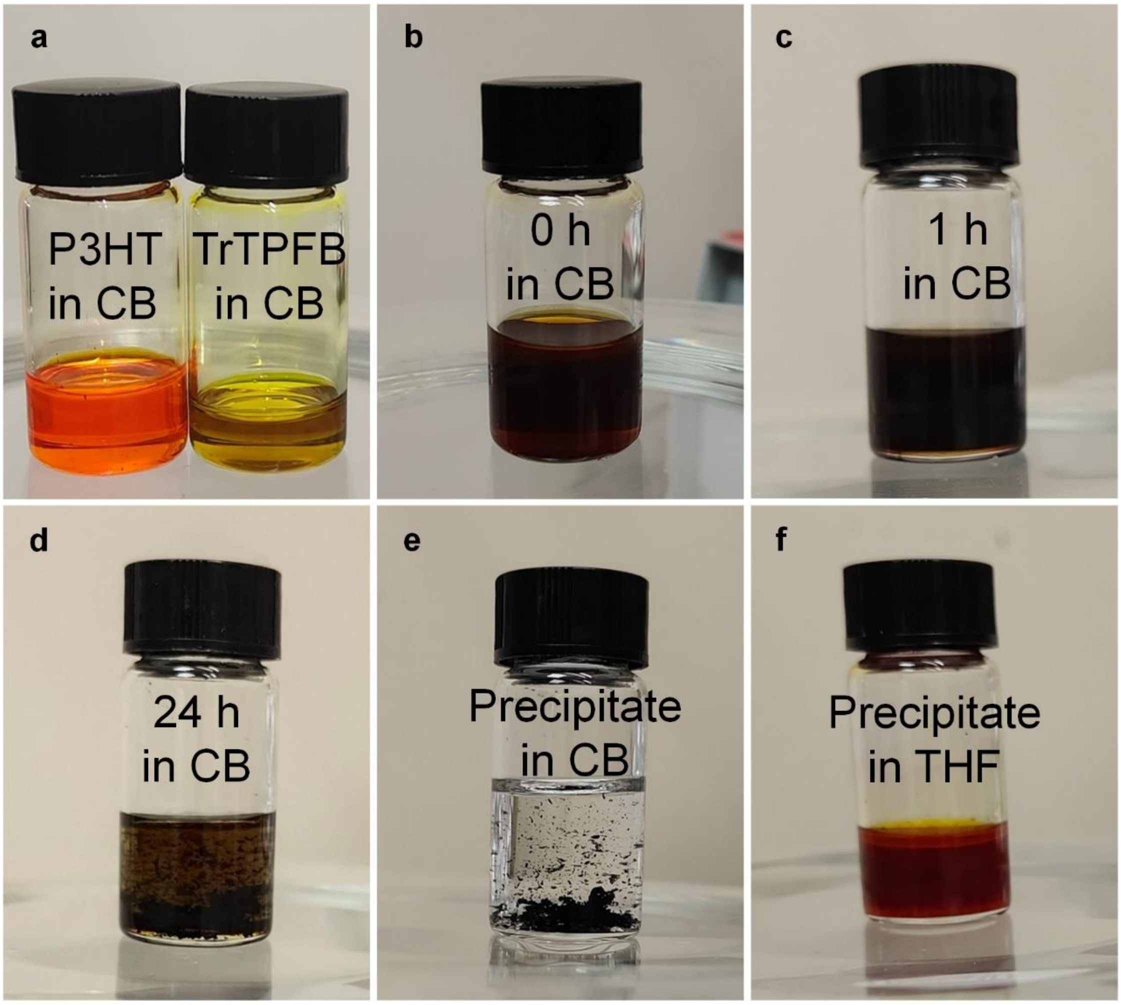

Figure S10. Characterization of the precipitations produced by doping P3HT with TrTPFB. (a) The pristine P3HT and TrTPFB solutions in CB. The change of over 100 mol% TrTPFB/P3HT blend solutions for (b) 0 h, (c) 1 h and (d) 24 h. (e) The precipitate obtained by centrifugation after washing three times with CB. (f) The re-dissolved precipitate in THF at 60 ℃.

For ^{1}H NMR characterizations of doped P3HT, it should be noted that we used the precipitates from TrTPFB-doped P3HT solution in CB. As seen in Figure S10, precipitation appears almost instantly when 100 mol% TrTPFB was mixed with P3HT

and the mixture showed apparent color changes. A lot of precipitates were obtained as the mixture was placed still for 24 hours. These precipitates could be separated and purified by centrifugation-washing with CB, and they were found to be highly conductive by electrical test with multimeter, which suggests they are doped P3HT. The precipitates can be dissolved in THF when heated at 60 ℃ (Figure S3f), making NMR characterization possible at elevated temperature.

We have also prepared samples of doped- OT_4 for NMR studies. In contrast, doped OT_4 is well soluble in organic solvents. The characterization of doped OT_4 was based on freshly prepared TrTPFB-OT_4 mixture in $CDCl_3$.

S4.2 Characterization of doped OT_4

High resolution electrospray ionization-mass spectrometry (ESI-MS) characterization can give very accurate molar mass (m/z) information of the molecules of interest. When the mixture of TrTPFB and OT_4 was characterized by high resolution ESI-MS, the products from the reaction of trityl cation and OT_4 could be clearly identified. The characteristic peaks and their corresponding species are:

908.4510: Doped or ionized (by ESI) Friedel-Crafts tritylation product, i.e., $[OT_4\text{-}CPh_3]^{\bullet+}$ (theoretical most abundant m/z = 908.4517, abundance = 100%).

909.4533: $[OT_4\text{-}CPh_3\text{-}H]^+$ (theoretical most abundant m/z = 909.4590, abundance = 100%), or doped or ionized (by ESI) Friedel-Crafts tritylation product, i.e., $[OT_4\text{-}CPh_3]^{\bullet+}$ (theoretical 2nd-most abundant m/z = 909.4550, abundance = 63.8%).

910.4535: The Wheland intermediate from the electrophilic attack of Ph_3C^+, i.e., $[OT_4\text{-}CPh_3\text{-}H]^+$ (theoretical 2nd-most abundant m/z = 910.4624, abundance = 63.8%), or doped or ionized (by ESI) Friedel-Crafts tritylation product, i.e., $[OT_4\text{-}CPh_3]^{\bullet+}$ (theoretical 3rd-most abundant m/z = 910.4584, abundance = 20.0%)

When matrix-assisted laser desorption-ionization time-of-flight (MALDI-TOF) mass spectrometry was employed, multi-tritylated OT_4 could be observed. This indicates that trityl cation is highly active toward those thiophene units.

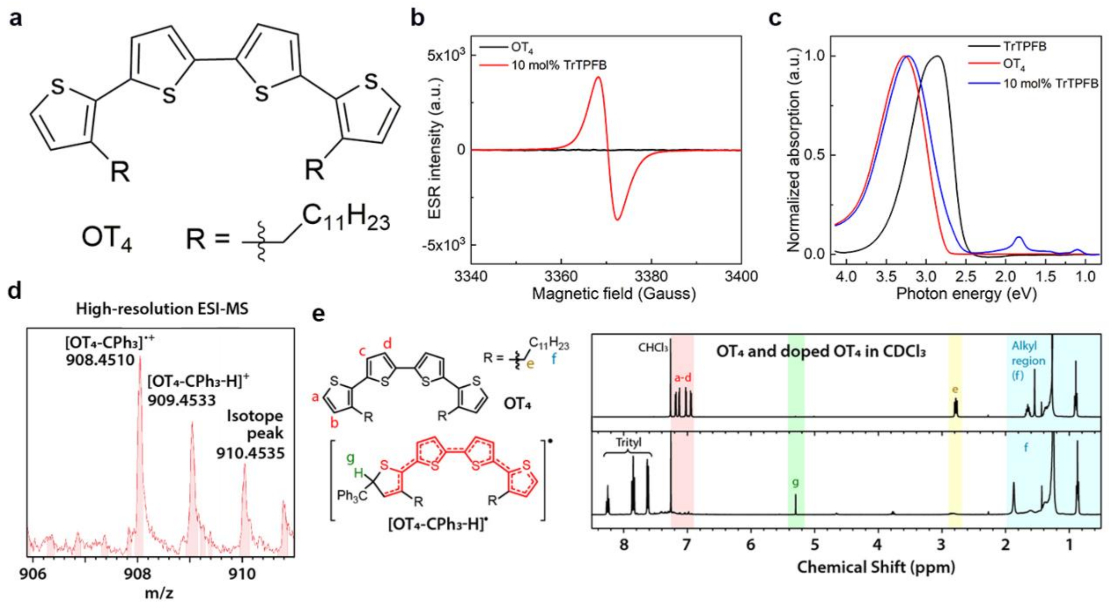

Figure S11. Characterization of TrTPFB-doped 3,3'''-Didodecyl-2,2':5',2'':5'',2'''-quaterthiophene (OT_4). (a) The molecular structure of OT_4. (b) The ESR of pristine and 10 mol% TrTPFB-doped OT_4. (c) The UV-vis-NIR absorption spectra of TrTPFB, OT_4 and 10 mol% TrTPFB-doped OT_4. (d) High-resolution ESI-MS analysis of TrTPFB-doped OT_4, a model oligomer of P3HT. Peaks corresponding to the tritylated OT_4 are clearly seen. (e) ^{1}H NMR characterization of OT_4 and TrTPFB-doped OT_4. Successful alkylation (as suggested by the peak of the proton colored in green) and formation of radicals are analogously observed.

It is worth mentioning that the 2 and 5 positions of thiophenes are the first choices for electrophilic attack, and the 3, 4 positions will only get substituted when the 2 and 5 positions are occupied. Thus, since the terminal thiophene units in OT_4 have unoccupied, more active 2- or 5-positions, the substitution will take place on the positions next to sulfur (as shown in Figure S11d).

S4.3 Further discussions on NMR characterization of doped OT_4

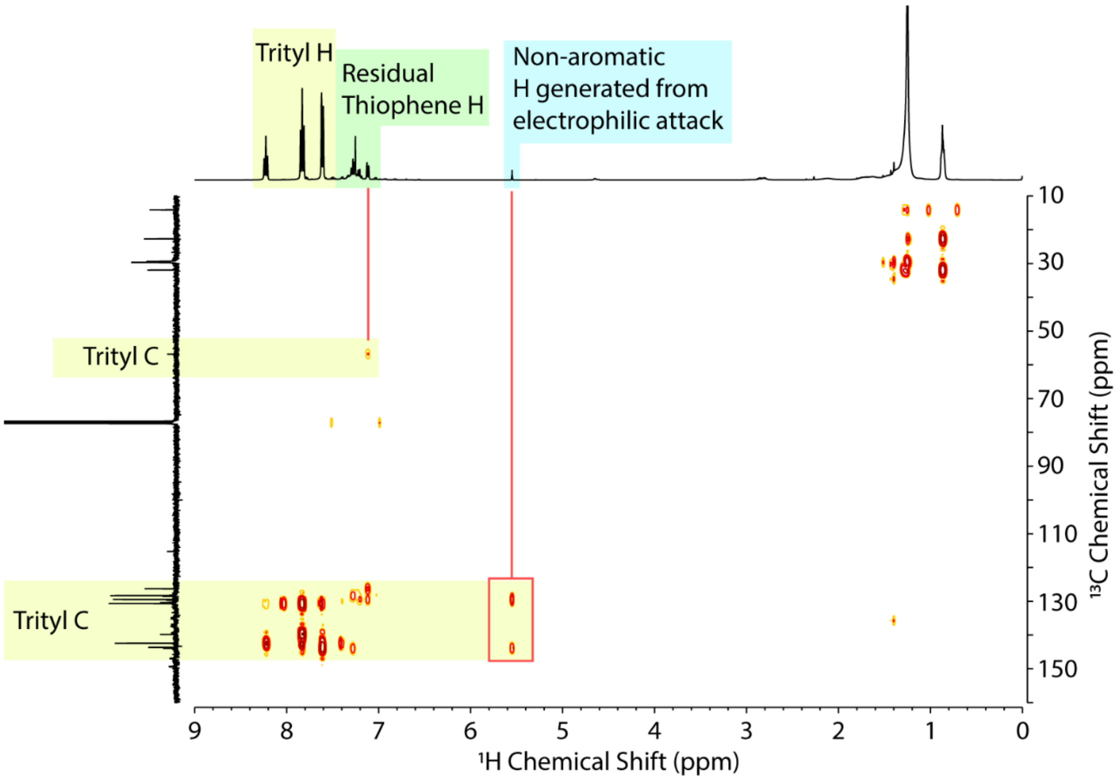

Figure S12. Result of a heteronuclear multiple bond correlation (HMBC) NMR characterization of TrTPFB-doped OT_4. ^{1}H NMR spectrum is on the horizontal axis and ^{13}C NMR spectrum is on the vertical axis. The cross-peaks, *i.e.*, the "red dots" denote the correlation between the peaks, indicating that the corresponding proton (as marked with red rectangle and lines) is at the close proximity (separated by 2 or 3 bonds) of the corresponding carbon atoms (as marked with yellow background). The appearance of the cross-peaks is a solid evidence for the covalent attachment of trityl group on thiophene units, and for that the peak at *ca.* 5.5 ppm is indeed assigned to the non-aromatic proton formed through the electrophilic attack of TrTPFB (green proton/peak in Figure 2c).

S5. Further discussions on the doping mechanism of Brønsted acids, TrTPFB and other electrophiles

S5.1. The role of Wheland intermediate in the acid doping process

P-doping of organic semiconductors (OSCs) by various Lewis acids and Brønsted acids has attracted wide attention as this doping process does not rely on any oxidation or reduction process, thus a dopant can be far more general in use compared to oxidants or reductants that can only be used on OSCs with matching HOMO/LUMO levels. Yurash *et al.* had conducted detailed studies that revealed the mechanism of this doping process, in which polarons form through oxidation-reduction reactions between chains after a portion of chains are protonated by the acids, i.e., electron transfer from a thiophene unit to a protonated thiophene unit[1]. In this reported doping mechanism, the protonated thiophene unit is directly responsible for the generation of polarons, serving as an oxidizer to fetch a single electron from another thiophene and generate two radicals. Such oxidation process is not unprecedented, as heterocyclic arenium ions can be highly effective oxidants as illustrated by the famous "blue bottle experiment", a classic chemistry demonstration used in laboratory courses as a general chemistry experiment.

In a chemical perspective, the protonation of thiophene ring, which is the key step in polaron generation for acid doping, is in fact an electrophilic attack process by the proton H^+. The protonated thiophene, an arenium ion, is usually called the Wheland intermediate. As the name suggests, Wheland intermediates are unstable species, subject to fast re-aromatization through deprotonation[2]. This is applicable to other aromatic system as well, and is a well-known reversible process. For example, deuterated benzene (C_6D_6) can be transformed to C_6H_6 by treating it with strong acid. This reversibility is the key reason responsible for the low doping efficiency of Lewis acids or Brønsted acids: because the arenium ion is in a very fast equilibrium with H^+ and free aromatic ring (like a benzene or thiophene unit), and its energy is much higher than the free aromatic ring because of the loss of aromaticity, the effective concentration of arenium ion is low. In the case of P3HT, this means the population of protonated thiophene unit, alternatively called the arenium ion, the thiophenium ion or the Wheland intermediate, is low, and thus the polaron generation caused by electron transfer from a thiophene unit to a protonated thiophene unit is difficult.

With the above mechanism, it can be expected that stronger acids, which can effectively provide more H^+, will promote the generation of the Wheland intermediate and therefore the generation of polarons. We have indeed observed this phenomenon. By doping P3HT films with Brønsted acids of different acidity, we clearly observed the difference (Figure S1): the stronger acids, HCl (pK_a = −2.2), p-TSA•H_2O (pK_a = −1.7) and TFA (pK_a = −0.3) showed significantly higher doping efficiency compared to p-TA (pK_a = 4.2). At low doping ratio, the conductivity of the doped polymer was well correlated with the dopant's pKa value (the lower the better). At higher doping ratio, the trend became complicated, likely a result of "solubility" difference of these acids in polymer matrix, and difference in counterion mobility. The report of DMSO-HBr doping system actually provided a complexation-based solution for this issue[3], but the "solubility" and mobility issue remains a general problem with acid doping method, namely strong acids are inherently highly polar and are unlikely to be well miscible with OSCs. That is, effectively, another kinetic barrier for the formation of Wheland intermediates. Moreover, the generation of proton by Lewis acids relies on the acids' hydrolysis, and such multi-component system built on hard-to-control factors, *i.e.*, residual water in OSC materials.

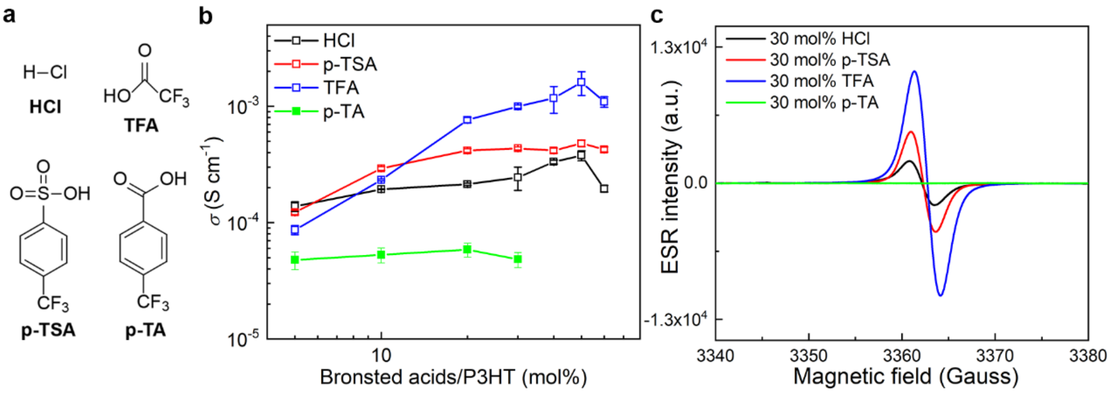

Figure S13. Characterization of P3HT films doped with different Brønsted acids. (a) The chemical structure of the Brønsted acids. (b) The conductivity of P3HT films doped with three Bronsted acids as a function of the doping concentration. (c) The ESR of P3HT films doped with the Brønsted acids at 30 mol%.

S5.2. Rational design for the stabilization of the Wheland intermediate

To stabilize the Wheland intermediate, a larger conjugated system may be implemented to stabilize the charged arenium species through resonance to compensate for the energy penalty from loss of aromaticity upon electrophilic attack. The stabilization effect can be so intense that the arenium species can become stable compounds (see Figure S14b). Fortunately, those OSC polymers always have large conjugated systems, which are by nature the basic requirement for achieving conductivity. Indeed, this is the very reason that OSCs such as P3HT can be doped by strong acids: trace amount of metastable arenium ion from the electrophilic attack of H^+ is possible (but difficult) because of the stabilization effect from the extended conjugated system.

When H^+ is replaced with other electrophilic reagents such as acylium ions or alkylium ions, electrophilic substitution on the arene ring will take place, presenting the well-known Friedel-Crafts reaction, one of the earliest name reactions in the history of organic chemistry. For example, monomeric thiophene reacts with TrTPFB and yields trityl thiophene, and the Wheland intermediate could not be isolated. However, when the conjugation system is larger, the Friedel-Crafts reaction may stop at the Wheland intermediate stage, as the stage is stabilized by both the large conjugation system and the steric effect from the substituent (provided by the corresponding electrophilic reagent). The root for such steric hindrance-related stabilization effect is explained in Figure S14a: the large substituent (in our case, the trityl group) “locks” the Wheland intermediate to a conformation that does not allow the dissociation of H^+, blocking the path for the generation of the corresponding Friedel-Crafts product. Thus, a higher population of arenium ion (the Wheland intermediate) can be obtained, which can serve as the true dopant for the OSC. This strategy is proved to be successful in both P3HT and OT_4 (tetrameric thiophene), efficiently triggering the formation of radicals and grant conductivity to the products.

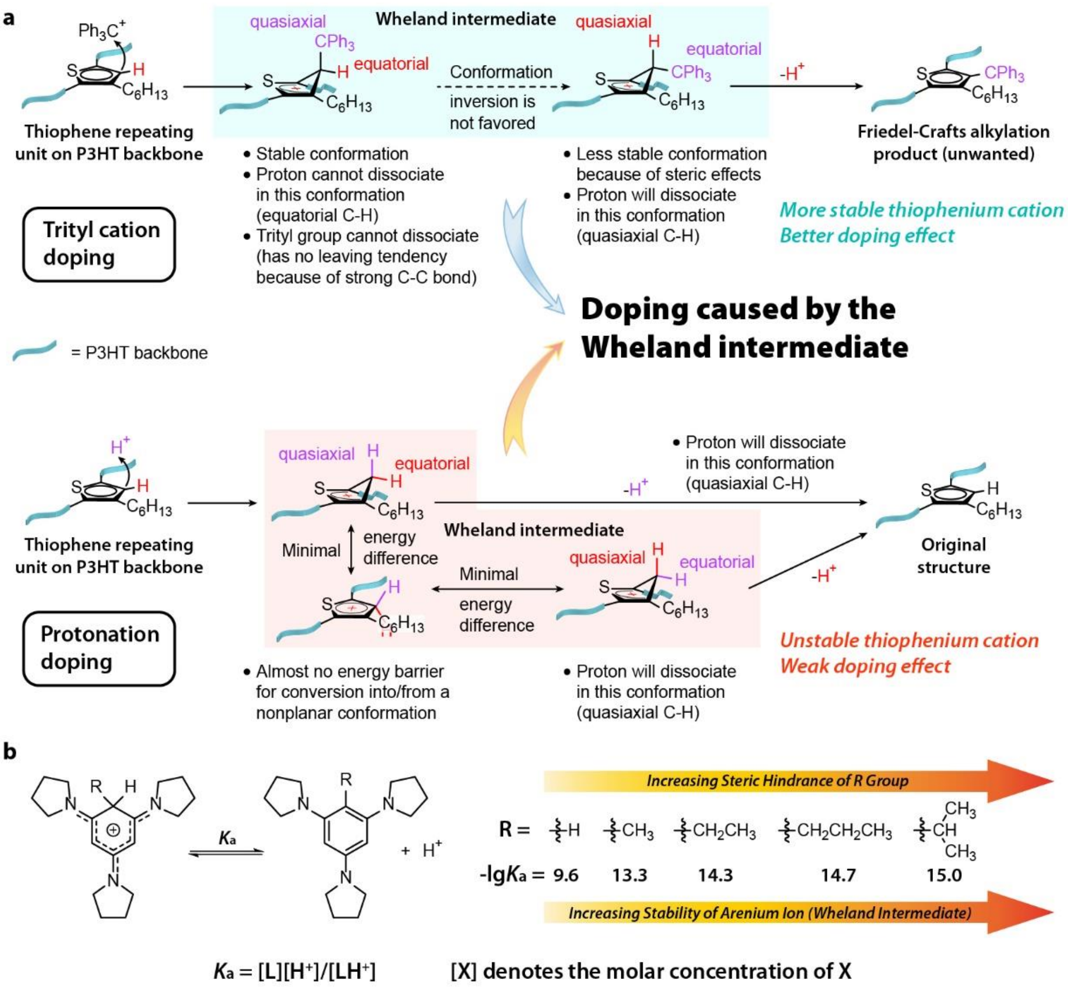

Figure S14. Mechanistic illustration of stabilized Wheland intermediate using a bulky substituent (trityl). (a) Conformational analysis of the arenium ions formed from alkylation (using trityl cation, top) or from protonation (bottom). Structures in the cyan or red backgrounds illustrate the conformations of the Wheland intermediates formed upon reacting with Ph_3C^+ or H^+. It should be noted that the hydrogen atom must be aligned with the empty *p* orbital of the π system in order to dissociate (as in an E1 elimination). This alignment is only possible when the hydrogen atom is at the quasiaxial position (pointing up). However, since the conformation with $-CPh_3$ at quasiaxial position is much more stable, proton elimination is largely prevented by a kinetic barrier. This makes the trityl substituted Wheland intermediate (thiophenium) much more stable, increasing its population and consequently giving a much higher efficiency in doping P3HT. (b) A previously reported structure-acidity relationship for a six-membered-ring arenium system[4]. The tendency for proton dissociation becomes lower as more sterically hindered R is used.

S5.3. Extension of the doping mechanism to other electrophiles

Significant doping effect for other electrophiles, such as nitronium tetrafluoroborate and diphenyliodium tetrakis(pentafluorophenyl)borate, was also observed when they were mixed with P3HT (see Figure S15). These compounds are also expected to undergo fast electrophilic attack on electron-rich aromatic systems like thiophene, generating Wheland intermediates responsible for effective doping. N-Bromosuccinimide (NBS), a Br^+ provider, showed only weak doping effect (see Figure S16), due presumably to bromine atom's small size and consequent inability in providing enough steric stabilization effect for the corresponding Wheland intermediate. These results, in another perspective, support that TrTPFB dopes P3HT through electrophilic attack.

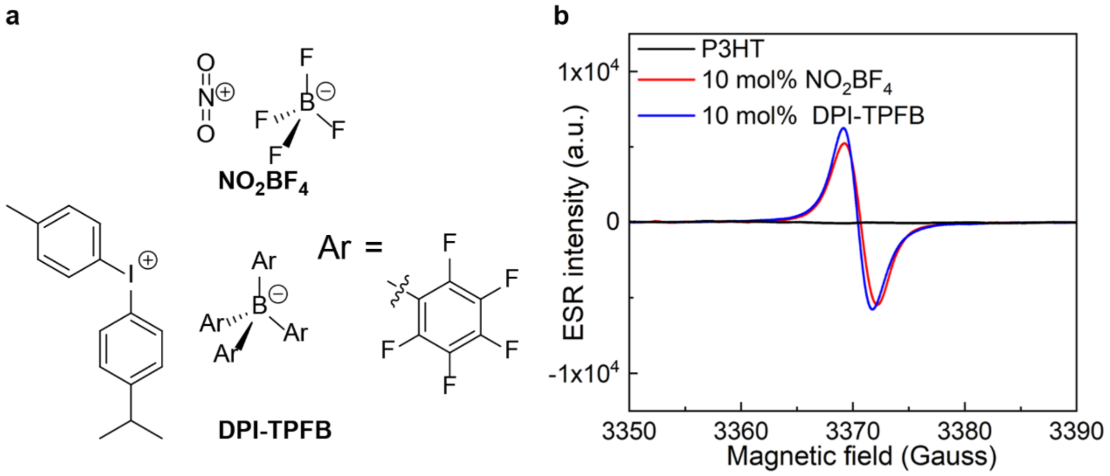

Figure S15. Characterization of P3HT doped with Nitronium tetrafluoroborate (NO_2BF_4) and 4-Isopropyl-4'-methyldiphenyliodonium Tetrakis(pentafluorophenyl)borate (DPI-TPFB). (a) The molecule structure of NO_2BF_4 and DPI-TPFB. (b) The ESR of pristine P3HT, NO_2BF_4- and DPI-TPFB-doped P3HT (10 mol%).

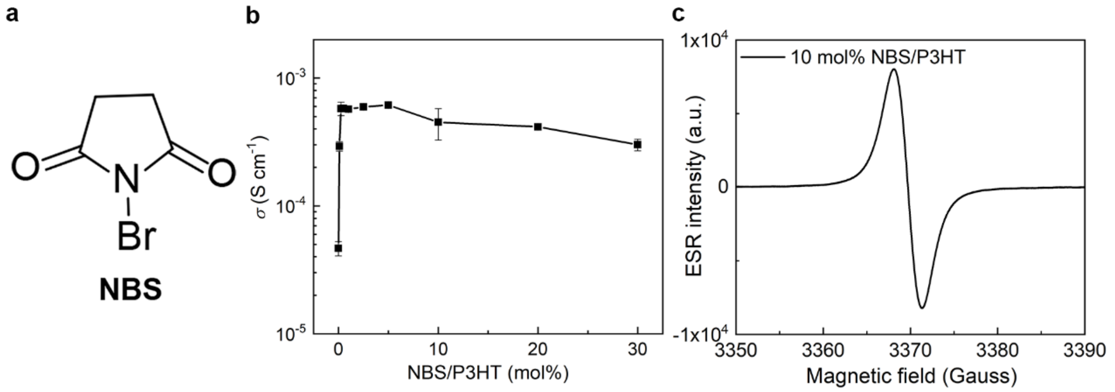

Figure S16. Characterization of P3HT films doped with NBS. (a) The chemical structure of NBS. (b) The conductivity of P3HT films doped with NBS as a function of the doping concentration. (c) The ESR of P3HT films doped with NBS at 10 mol%.

S6. Characterization of dopant polaron yielding efficiency and doping efficiency

S6.1 Absorption spectra characterizations

To begin with, F_4TCNQ was taken as a reference dopant to dope P3HT solutions (chlorobenzene as solvent), whose absorption as a function of doping ratio is shown in Figure 3c. A broad absorption in the range 1.1 - 1.75 eV is observed upon doping. On the basis of previous studies, this absorption feature can be ascribed to the dopant-induced polaron on the P3HT backbone. In addition, as reported in literatures, the F_4TCNQ-doped P3HT spectrum shows the characteristic double peak of the F_4TCNQ radical anion at 1.43 and 1.65 eV, suggesting that integer charge transfer occurs between P3HT and F_4TCNQ[5]. For comparison, the TrTPFB doped solutions show emergence of two absorption peaks at about 1.3 and 1.6 eV, which again are attributed to formation of polarons. Aside from these absorptions, there also exists a broad absorption peak around 2.2 eV, which was assigned as the absorption of neutral ordered P3HT aggregates[5c, 5e]. Based on the above knowledge, we performed Gaussian fit to the experimentally measured absorption into separation absorption designated to each species. The results are shown in the Figure S17 and Table S2.

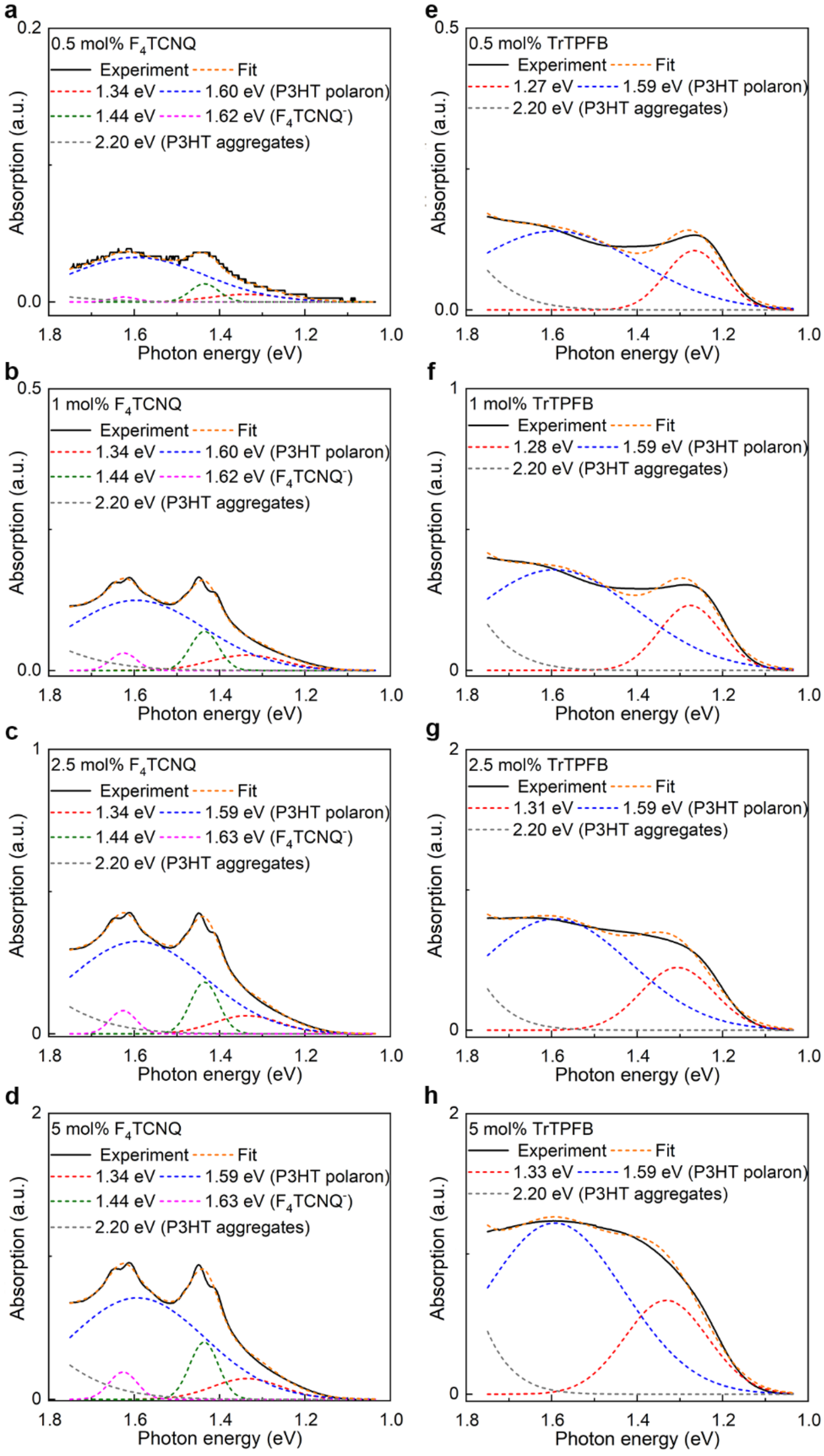

Figure S17. The decomposition of the UV-vis-NIR absorption spectra in the range of 1.3 eV to 1.75 eV for F_4TCNQ- and TrTPFB-doped P3HT solutions. The F_4TCNQ-doped P3HT at concentrations of (a) 0.5 mol%, (b) 1 mol%, (c) 2.5 mol%

and (d) 5 mol%. The TrTPFB-doped P3HT at concentrations of (e) 0.5 mol%, (f) 1 mol%, (g) 2.5 mol% and (h) 5 mol%.

Table S2. Details of polaron peaks in doped P3HT solutions extracted from fitting the experimental absorption.

**F_4TCNQ**	**0.5 mol%**		**1 mol%**		**2.5 mol%**		**5 mol%**	
Position (eV)	1.34	1.60	1.34	1.60	1.34	1.59	1.34	1.59
FWHM (eV)	0.20	0.38	0.20	0.38	0.19	0.38	0.19	0.37
Intensity (mol^{-1} L)	5.56	32.56	27.29	124.48	63.79	324.53	144.75	708.92
**TrTPFB**	**0.5 mol%**		**1 mol%**		**2.5 mol%**		**5 mol%**	
Position (eV)	1.27	1.59	1.28	1.59	1.31	1.59	1.33	1.59
FWHM (eV)	0.16	0.46	0.17	0.45	0.20	0.42	0.23	0.38
Intensity (mol^{-1} L)	105.23	139.87	230.63	357.28	447.00	792.92	669.23	1220.70

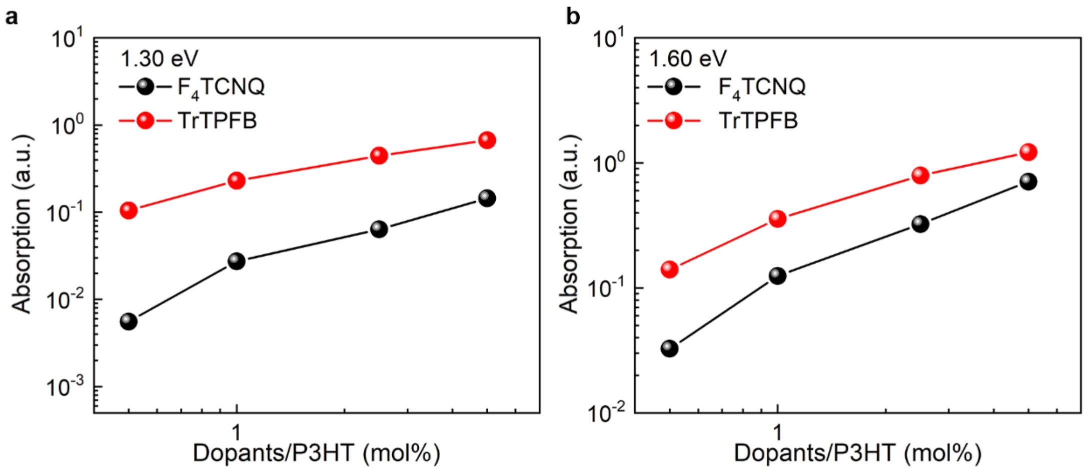

Figure S18. The F_4TCNQ-doped P3HT spectrum shows the characteristic double peak of the F_4TCNQ radical anion at 1.43 and 1.63 eV, suggesting that integer charge transfer occurs between P3HT and F_4TCNQ[5]. The polarons of P3HT has two absorption peaks located at 1.30 and 1.60 eV. By decomposing the absorption with the known information, we obtained the amplitude of each polaron peak. The intensity of the P3HT polaron absorption peak at (a) 1.30 eV and (b) 1.60 eV obtained from the decomposed spectrum as a function of dopant concentration.

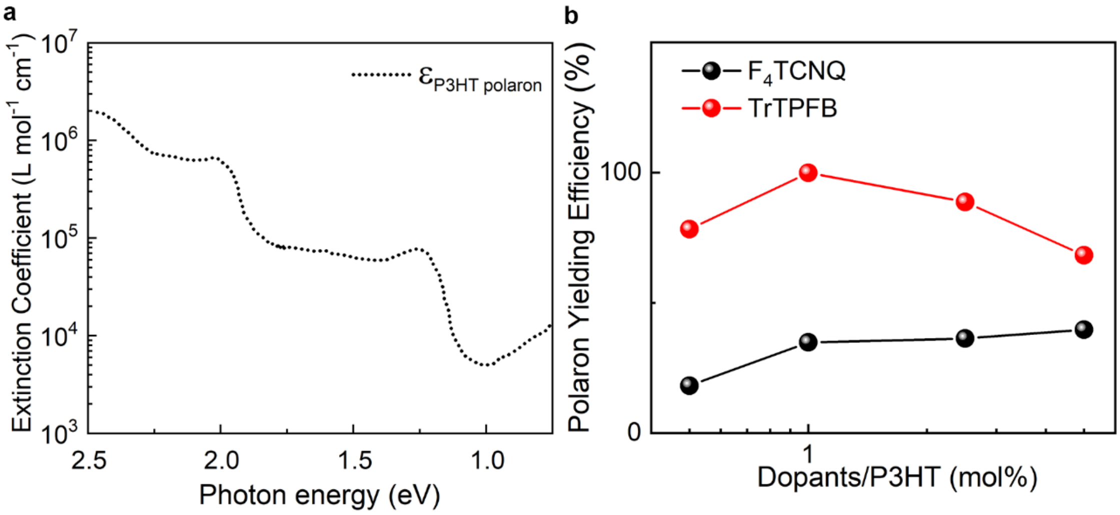

Figure S19. Extraction of the polaron yielding efficiency from the UV-vis-NIR absorption spectra of doped solutions. (a) The extinction coefficient of P3HT polaron obtained from reference[5d]. (b) The polaron yielding efficiency of different doping concentrations extracted from the P3HT polaron absorption peak.

The values in Figure S19b were obtained according to equation S1 reported in the literature[5d]:

$$(OD/d) = \varepsilon_{P3HT\ polaron} \times c \times X \quad \text{(S1)}$$

where OD/d is the normalized absorbance of the path length (d), ε is the molar extinction coefficient of the absorbing substance, c is the dopant concentration, and X is the polaron yielding efficiency.

S6.2 ESR characterizations

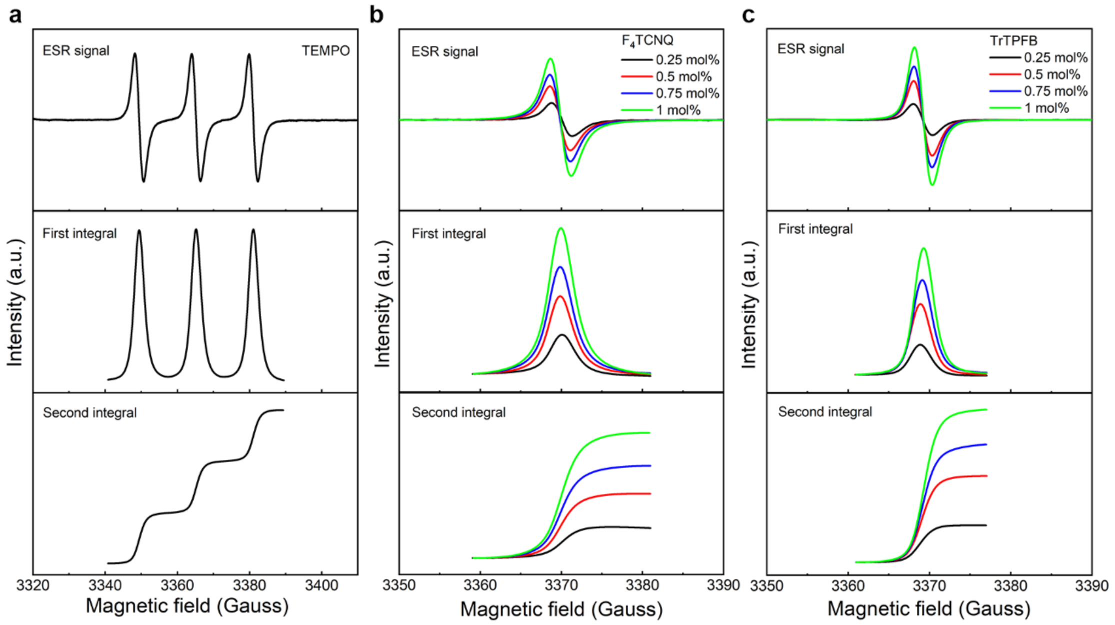

Figure S20. The extraction of spin numbers by quantitative ESR. The ESR signal, its first integral and second integral of (a) TEMPO, (b) F_4TCNQ- and (c) TrTPFB-doped P3HT films. As a reference sample, one TEMPO molecule corresponds to one spin. The spin numbers of the doped samples were obtained by comparing their second integral of ESR signals with that of the standard sample, according to the equation: $spins = \frac{second\ integral}{calibration\ factor*(\sqrt{microwave\ power}*B_{mod}*Q*nB*S*(S+1))}$, where B_{mod} is the modulation intensity of magnetic field, nB the Boltzmann population difference, Q the quality factor of the cavity, and S=1/2 for a doublet state.[6]

One notable thing for the extraction of polaron density is that we assume only polarons (radical cations) contribute to the ESR signal. Such assumption is based on previous studies claiming that the neutral radicals shown in Figure 2b reacts to release H_2. Besides, we noted that the polaron yielding efficiency obtained by absorption spectra for TrTPFB is about 100 % at doping levels lower than 1 mol%, being consistent with the values achieved by ESR method, which indicates reasonability of our assumption.

S6.3 Mott-Schottky analysis

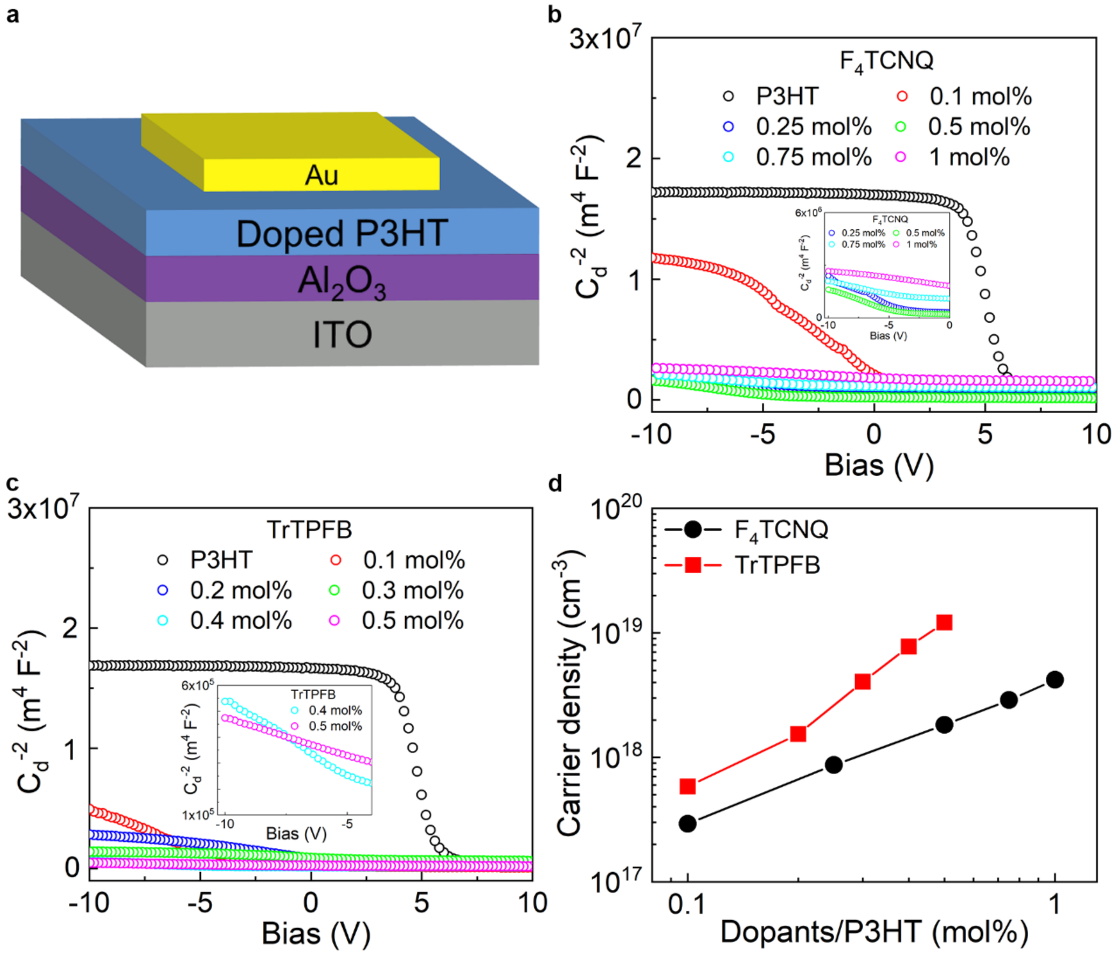

Figure S21. The extraction of charge carrier density by Mott-Schottky analysis. (a) The structure diagram of MIS diode. Mott-Schottky plots of (b) F_4TCNQ and (c) TrTPFB-doped P3HT films at different doping ratios. (d) The carrier density extracted from the Mott-Schottky plots.

S7. Characterization of thermoelectric performance

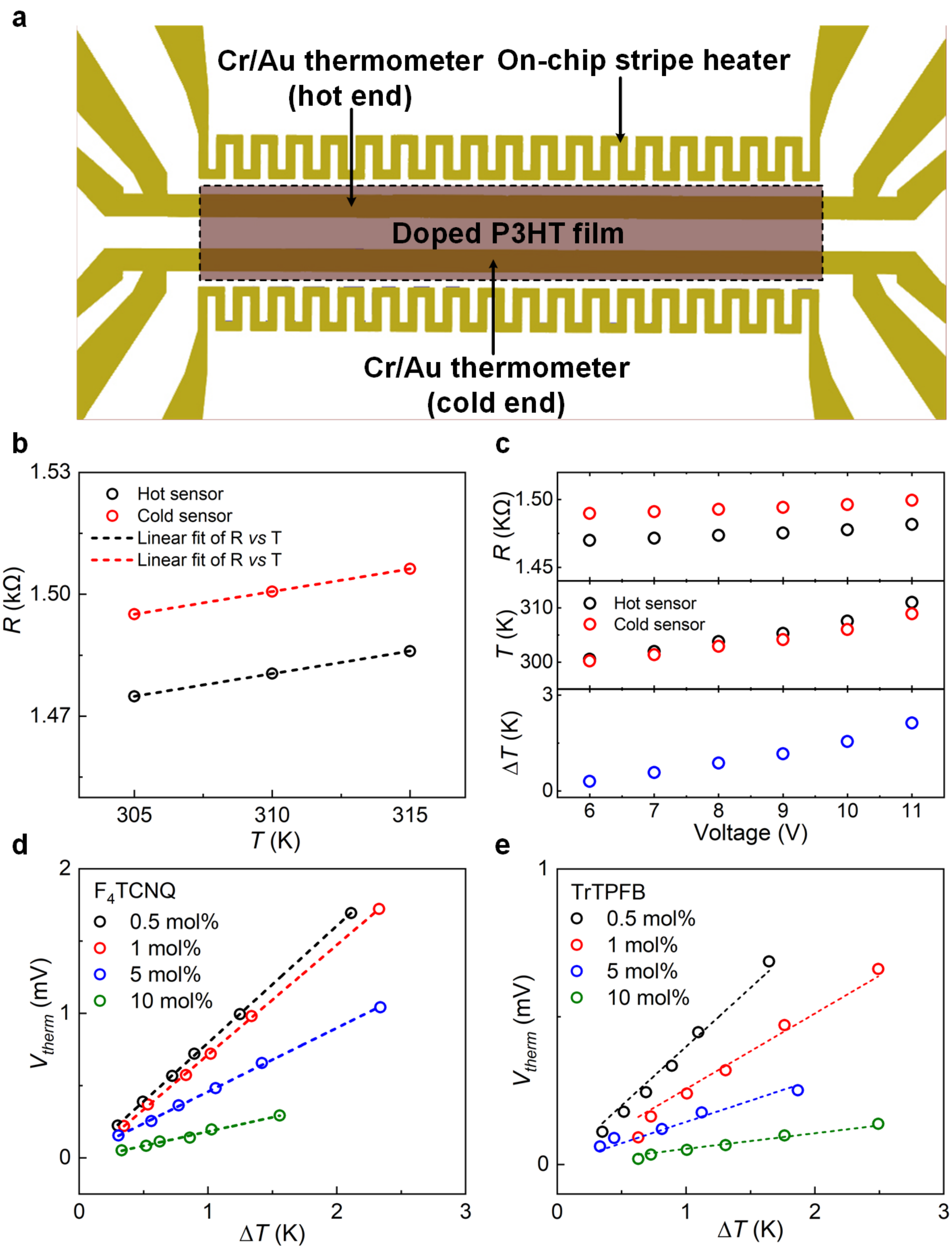

Figure S22. The thermoelectric characterization of P3HT films doped with F_4TCNQ and TrTPFB. (a) The schematic of device structure used to measure Seebeck coefficient. (b) The temperature coefficient of resistance (TCR) is the slop of the resistance versus temperature. (c) The estimated temperature of hot and cold sensors obtained using the equation S2-5. The thermal voltage of the films doped with two

dopants as a function of the temperature difference at different doping concentrations: (d) F_4TCNQ as dopant and (e) TrTPFB as dopant.

$$T_{hot} = 305 + \frac{R_{hot} - R_{hot,305K}}{\mathrm{TCR}} \tag{S2}$$

$$T_{cold} = 305 + \frac{R_{cold} - R_{cold,305K}}{\mathrm{TCR}} \tag{S3}$$

$$\Delta T = R_{hot} - R_{cold} \tag{S4}$$

$$S = \lim_{\Delta T \to 0} \frac{\Delta V}{\Delta T} \tag{S5}$$

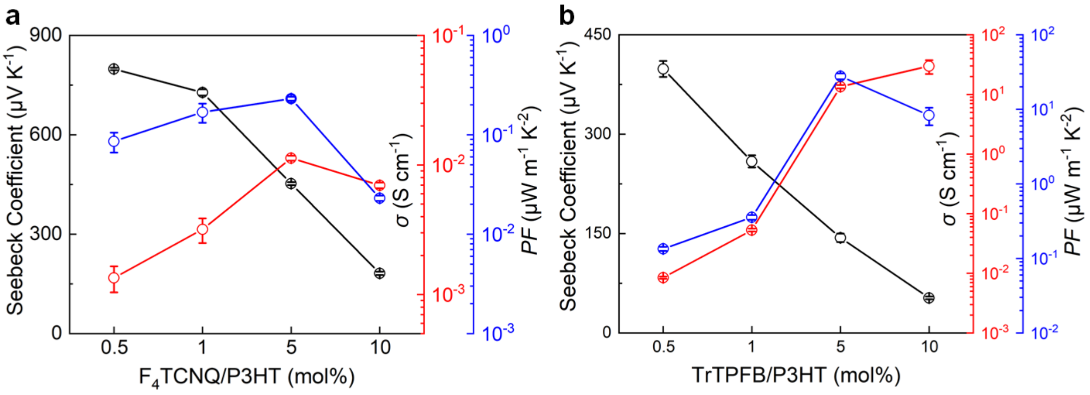

Figure S23. The thermoelectric performance of doped P3HT films. The Seebeck coefficients, conductivity and power factor of P3HT doped with (a) F_4TCNQ and (b) TrTPFB at variable doping concentrations.

The *S* value of F_4TCNQ-doped films varies from 800 μV K^{-1} to 180 μV K^{-1} as the doping ratio changes from 0.5 mol% to 10 mol%. The maximum *PF* values we have achieved in F_4TCNQ doped films are on the order of 0.23 μW $m^{-1}K^{-2}$. These results regarding to thermoelectric performance of F_4TCNQ-doped P3HT films are quite consistent with previous reports[7], and therefore the results about the thermoelectric performance of TrTPFB-doped P3HT should be reliable.

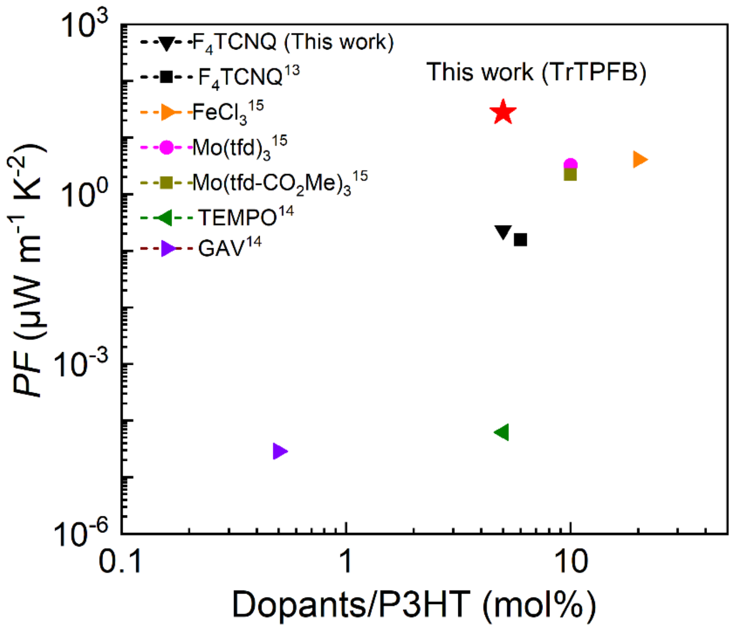

Figure S24. The power factor of spin-coated P3HT films doped by TrTPFB and other dopants from references using the solution doping method[8].

S8. Stability of TrTPFB-doped P3HT films

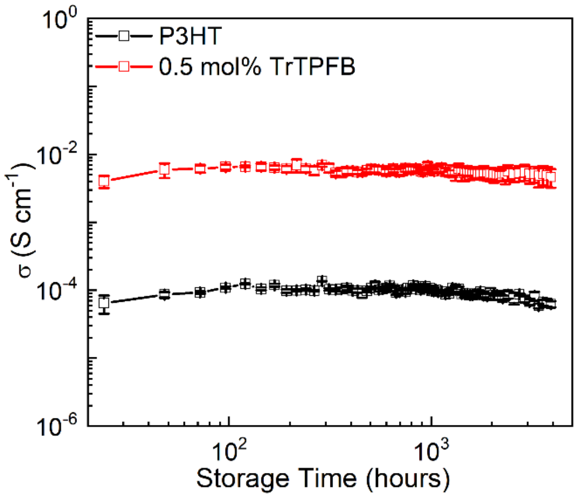

Figure S25. The conductivity of P3HT films doped with TrTPFB in ambient atmosphere as a function of storage time (RH = 20 ~ 40 %).

REFERENCES

[1] B. Yurash, D. X. Cao, V. V. Brus, D. Leifert, M. Wang, A. Dixon, M. Seifrid, A. E. Mansour, D. Lungwitz, T. Liu, *Nat. Mater.* **2019**, 18, 1327.

[2] a) I. Galve, R. Ondoño, C. de Rocafiguera, R. Puig de la Bellacasa, X. Batllori, C. Puigjaner, M. Font-Bardia, O. Vallcorba, J. Teixidó, J. I. Borrell, *Org. Biomol. Chem.* **2020**, 18, 9810; b) S. M. Hubig, J. K. Kochi, *J. Am. Chem. Soc.* **2000**, 122, 8279.

[3] N. Sakai, R. Warren, F. Zhang, S. Nayak, J. Liu, S. V. Kesava, Y.-H. Lin, H. S. Biswal, X. Lin, C. Grovenor, T. Malinauskas, A. Basu, T. D. Anthopoulos, V. Getautis, A. Kahn, M. Riede, P. K. Nayak, H. J. Snaith, *Nat. Mater.* **2021**, 20, 1248.

[4] F. Effenberger, F. Reisinger, K. H. Schoenwaelder, P. Baeuerle, J. J. Stezowski, K. H. Jogun, K. Schoellkopf, W. D. Stohrer, *J. Am. Chem. Soc.* **1987**, 109, 882.

[5] a) P. Pingel, D. Neher, *Phys. Rev. B* **2013**, 87; b) C. Wang, D. T. Duong, K. Vandewal, J. Rivnay, A. Salleo, *Phys. Rev. B* **2015**, 91; c) L. Müller, D. Nanova, T. Glaser, S. Beck, A. Pucci, A. K. Kast, R. R. Schröder, E. Mankel, P. Pingel, D. Neher, W. Kowalsky, R. Lovrincic, *Chem. Mater.* **2016**, 28, 4432; d) M. Arvind, C. E. Tait, M. Guerrini, J. Krumland, A. M. Valencia, C. Cocchi, A. E. Mansour, N. Koch, S. Barlow, S. R. Marder, J. Behrends, D. Neher, *J. Phys. Chem. B* **2020**, 124, 7694; e) A. E. Mansour, D. Lungwitz, T. Schultz, M. Arvind, A. M. Valencia, C. Cocchi, A. Opitz, D. Neher, N. Koch, *J. Mater. Chem. C* **2020**, 8, 2870.

[6] P. Pingel, M. Arvind, L. Kölln, R. Steyrleuthner, F. Kraffert, J. Behrends, S. Janietz, D. Neher, *Adv. Electron. Mater.* **2016**, 2, 1600204.

[7] G. Zuo, O. Andersson, H. Abdalla, M. Kemerink, *Appl. Phys. Lett.* **2018**, 112, 083303.

[8] a) Y. Zou, D. Huang, Q. Meng, C.-a. Di, D. Zhu, *Org. Electron.* **2018**, 56, 125; b) Z. Liang, Y. Zhang, M. Souri, X. Luo, Alex M. Boehm, R. Li, Y. Zhang, T. Wang, D.-Y. Kim, J. Mei, S. R. Marder, K. R. Graham, *J. Mater. Chem. A* **2018**, 6, 16495; c) E. P. Tomlinson, S. Mukherjee, B. W. Boudouris, *Org. Electron.* **2017**, 51, 243.
